# The Efficacy and Safety of the Zhuyun Formula and Auricular Acupressure for the Infertile Women with Recurrent Implantation Failure: A Randomized Controlled Trial

**DOI:** 10.1155/2022/5274638

**Published:** 2022-10-11

**Authors:** Hang Zhou, Xiaoyan Zheng, Wanting Xia, Qianhong Ma, Jinmei Li, Qian Zeng, Jinzhu Huang

**Affiliations:** ^1^School of Basic Medicine, Chengdu University of Traditional Chinese Medicine, Chengdu 611137, China; ^2^Department of Gynecology, Hospital of Chengdu University of Traditional Chinese Medicine, Chengdu 610037, China; ^3^Acupuncture and Tuina School, Chengdu University of Traditional Chinese Medicine, Chengdu, 611137, China; ^4^Chengdu Xi'nan Gynecology Hospital, Chengdu, 610020, China; ^5^Department of Gynecology, West China Second University Hospital of Sichuan University, Chengdu 610000, China; ^6^Department of Gynecology, Zhumadian TCM Hospital, Zhumadian 463000, China; ^7^School of Nursing, Chengdu University of Traditional Chinese Medicine, Chengdu 611137, China

## Abstract

**Background:**

Recurrent implantation failure (RIF), a clinical disorder characterized by failure to achieve pregnancy after repeated (≥3) embryo transfer, is a challenge for reproductive demands worldwide. In our preliminary work, the Zhuyun formula (ZYF) with auricular acupressure, a complementary and alternative medicine (CAM) with a small sample size for RIF, can improve the clinical pregnancy rate (41.2% vs. 26.7%, treatment group vs. control group, *p* < 0.05). Based on the toxicological and pregnancy-related pharmacological analysis of ZYF for RIF, the T-cell receptor signaling pathway might be involved in the pharmacological activity. This study aimed at evaluating the efficacy and safety of the CAM therapy according to pregnancy outcomes and maternal and child health and investigating the changes of T-helper (Th) cells in the peripheral blood of unexplained RIF women.

**Materials and Methods:**

We conducted a prospective, two-arms, randomized, nonblinded study. All eligible women were randomly assigned to the treatment group (TG) and the control group (CG) according to a computer-generated randomization list in sealed opaque envelopes. Blood samples were collected from the two groups, and serum Th1, Th2, and Treg were detected by flow cytometry. The cytokines were detected by an enzyme-linked immunosorbent assay (ELISA). The TG was administrated with ZYF and auricular acupressure for three months before ovarian stimulation, while the control group was on a waiting list for the same period. The primary outcome was CPR. The second outcomes were the serum levels of immune parameters. For the safety evaluation, the perinatal outcomes of maternal and child were obtained by follow-up. Post-hoc sensitivity analyses were performed to assess the effect of missing data.

**Results:**

One hundred and twenty-three women were randomized into the TG (*n* = 62) and CG (*n* = 61). The CPR was increased significantly in the TG (45.2%) than CG (26.2%) (*p* = 0.029). Twenty blood samples were collected, and the Th2/Th1 and Treg expression level was significantly higher in the TG than in the CG. IL-2, IL-10, and Foxp3 were higher significantly in the TG than in the CG. The maternal and child perinatal outcomes were not significantly different between the two groups.

**Conclusions:**

The ZYF with auricular acupressure was effective and safe in improving the pregnancy outcomes of RIF. It might be related to balancing the level of cytokines related to the immune tolerance of the maternal-fetal interface to protect the embryo from the maternal immune system. Clinical Trial Registration: Clinical Trial Registry; date: 14/Dec/2013; no. NCT03078205.

## 1. Introduction

Numerous progress in reproductive technology has significantly increased the opportunity of being parents for infertile couples [1]. However, a new challenge has emerged: recurrent implantation failure (RIF) [2]. Although there is no universally agreed-upon definition [3], RIF is often defined as the failure to achieve a clinical pregnancy after the repeated transfer (≥3) of more than four good-quality embryos under the age of 40 years [3, 4]. RIF is still one of the most challenging clinical dilemmas worldwide since the low clinical pregnancy rate (CPR) of in vitro fertilization and embryo transfer (IVF-ET). There is 10% incidence of RIF patients among the IVF-ET[2, 5, 6]. Currently, the etiology search and treatment of RIF mainly focus on the following three aspects: 1) maternal-fetal factors mainly including reproductive organ lesions, endometrial receptivity, and thrombotic diseases; 2) embryo factors mainly include chromosomal abnormalities, zona pellucida sclerosis, embryo culture, and transfer, and the malefactors; and 3) the immune factors, which gained increased attention recently, such as immune cells including T-helper cells (Th1), Th2, Treg, Th17, mononuclear macrophages, and natural killer cells, which are all important cells involved in the process of implantation. Autoimmune disease antiphospholipid antibodies and antinuclear antibodies have been confirmed to have higher plasma concentrations in patients with recurrent implantation failure than in healthy women of childbearing age, especially for the unexplained RIF [7–9].

Traditional Chinese medicine (TCM) therapy with a rich theoretical basis and clinical application for treating infertility includes Chinese herbs [10], acupuncture [11, 12], moxibustion [13], and taichi (exercise), which have been widely and increasingly applied in the clinic as a complementary and alternative medicine (CAM) [14]. Kidney deficiency, Qi stagnation, and blood stasis play crucial parts in infertility pathogenesis. The Zhuyun formula (ZYF) is composed of Wuzi Yanzong Wan (WZYZW) [15, 16], Sini San (SNS) [17], and Siwu decoction (SWD), which are the most classic recipes for kidney nourishment, liver regulation, and blood circulation. The components and molecules of ZYF are shown in Table S1 and Figure S1. Auricular acupressure is also widely used for treating infertility, especially for decreasing distress [18]. We found the significant efficacy of a CAM therapy (ZYF combined with auricular acupressure) in the preliminary trial with a small sample size (*n* = 30, each group) for RIF underwent IVF-ET (CPR: 41.2% vs. 26.7%, treatment group vs. control group *p* < 0.05). Furthermore, based on the therapeutic efficacy, we performed network pharmacology of ZYF for the RIF target protein and biological regulation process to identify the therapeutic mechanism of ZYF on specific target proteins involved in RIF (Tables S2 and S3). We also found out that the biological regulation process of immune tolerance during embryo implantation (ET) and the T-cell receptor signaling pathway (Tables S4 and S5), especially, might be involved in the pharmacological activity of ZYF on RIF. However, reliable evidence supporting the efficacy and safety of the CAM therapy use during the IVF-ET is still lacking. Whether the CAM therapy affects postpartum recovery and normal development of newborns in the long term is also widely of concern for medical workers and families. Consequently, focusing on evaluating the effects on perinatal outcomes and long-term health among these infants should be complemented in the follow-up phase.

Accordingly, considering the particularity of the population (women of childbearing and who are pregnant) and safety concerns, we conducted the randomized, nonblinded study to evaluate the efficacy and safety of ZYF and auricular acupressure to reduce the selection bias. Our findings provided a scientific, effective, and systematic theory and contributed to improving the evidence regarding the safety of the ZYF during IVF-ET.

## 2. Materials and Methods

### 2.1. Sample Size

Combining our preliminary study, evidence from the systematic reviews, and clinical advice, we estimated that a 25% or greater increase in the proportion of clinical pregnancy will be clinically important. To obtain 80% power at a 5% significance level for a 2-sided test, we assume a proportion of 24.5% [19] clinical pregnancies in the control group and 49.5% clinical pregnancies in the treatment group. The minimal sample size calculated is 55 for each group. Considering about a 10% dropout rate, 121 participants in total are needed. The sample size was calculated by PASS 15.0 software.

### 2.2. Study Design

Women of childbearing age or pregnancy are special, and the placebo setting may increase the likelihood of declining enrollment. To reduce the placebo effect, objective outcomes were observed. Thus, the study was a nonblinded, single-center, prospective, randomized controlled trial conducted in the West China Second University Hospital, Sichuan University. All eligible patients collected in the study agreed to participate and signed an informed consent form. The study was approved by the ethics committees (2016KL-013). The authors registered the trial with Clinical-Trials.gov (NCT03078205). Details of the study design (Figure S2), rationale for the primary and secondary outcome measures, power analyses, and the statistical analysis plan are available in the protocol (Supplementary Materials 1). It complies with the guidelines prescribed by the Consolidated Standards of Reporting Trials (CONSORT) checklist (Supplementary Materials 2).

### 2.3. Patient Recruitment

Participants diagnosed with RIF [5] and aged between 20 and 39 years who were undergoing ART will be included. Women who were planning cycles of preimplantation genetic testing (PGT), preimplantation genetic screening (PGS), reproductive malformation, and reproductive inflammation, as well as those with a diagnosis of congenital abnormality (such as a submucous myoma, intrauterine adhesion, or uterine malformation), autoimmunity, and endocrine disorders, and their husbands diagnosed with serious asthenozoospermia or oligospermia, were excluded from this trial.

### 2.4. Randomized and Masking

Women were recruited at the time of the diagnosis of RIF without remaining embryos, and the decision to undergo a new IVF cycle or intracytoplasmic sperm injection (ICSI) cycle and randomization occurred before the new menstrual cycle. Subjects were randomly allocated to the treatment group by a selection of the sealed envelope in the sequence based on a computer-generated list. Nurses did the randomization and the procedure was performed blinded to both the patients and to the clinician who performed the embryo transfer.

### 2.5. Intervention

In the treatment group, CAM treatment was utilized for three months before follicle stimulation, which included two kinds of interventions: ZYF and auricular acupuncture. ① Z YF was given three times daily, 200 ml per time. The decoction was composed of ZYF, which contained Tu Sizi (*Cuscuta chinensis Lam.*) 15 g, Fu Penzi (*Rubus idaeus L*.), Gou Qizi (*Lyciumchinense Mill.*) 10 g, Chai Hu (*Bupleurum chinense*) 10 g, Bai Shao (*Cynanchum otophyllum*) 15 g, Zhi Ke (*Poncirus trifoliata* (*L*.) *Raf*) 10 g, Dang Gui (*Angelica sinensis (Oliv.) Diels*) 10 g, Chuan Xiong (*Ligusticum chuanxiong Hort.*) 10g , Shu Dihuang (*Rehmannia glutinosa (Gaert.*) *Libosch. ex Fisch. et Mey.*) 10 g, and Zhi Gancao (*Glycyrrhiza uralensis Fisch.*) 5 g. All the ingredient herbs were extracted with boiled water to make an aqueous extract. The decoction can nourish the kidney essence, smooth the liver Qi, and circulate the blood. ② Auricular acupuncture: Small stainless needles for auricular treatment at the following points, Liver (CO_12_), Shenmen (TF_4_), Neifenmi (CO_18_), E (AT_1_), Nie (AT_2_), and Zhen (AT_3_) were used in the trial before IVF/ICSI for three months, once per week. All treatments were performed by the same well-trained examiner, in the same way. The details of composition, the source and the dosage of ZYF, and the location of auricular points are shown in Table S5 and Figure S3 in Supplementary Material 1.

In the control group, all participants were naturally waiting for 3 months before IVF/ICSI. For all subjects undergoing ART after three months, a standard long agonist protocol for ovarian stimulation was performed [20]. Egg retrieval, fertilization, and embryo transfer were determined by their treating clinician.

### 2.6. Outcomes

Information on demographics, fertility history, and health status were collected from subjects. To reduce the placebo efficacy of the CAM therapy as possible, we collected the objective outcomes in our study. The details of the definition and calculation of the outcomes were shown in the protocol (Supplementary Materials 1)

### 2.7. The Pregnancy Outcomes after IVF-ET

For the primary analysis, we first compared the proportion of women with clinical pregnancy in the two groups for all participants using relative risks (RRs) with 95% confidential difference (CI) with a hypothesis test for no effect. Clinical pregnancy is defined as the presence of at least one intrauterine gestational sac or fetal heartbeat confirmed by ultrasound, 4∼6 weeks after embryo transfer. The second outcomes were natural conception, ongoing pregnancy rate, and pregnancy loss (including biochemical miscarriage, clinical pregnancy loss, and ectopic pregnancy).

### 2.8. The Expression Levels of Th1 (IL-2, IFN-*γ*), Th2 (IL-4, IL-10), and Treg (Foxp3) in Peripheral Blood

To describe and analyze the benefit of CAM for unexplained RIF, the level of immune cells (Th1 (IL-2, IFN-*γ*), Th2 (IL-4, IL-10), and Treg (Foxp3)) was tested. Enrolled subjects diagnosed with unexplained RIF had serum levels checked. Serum levels of Th1 (IL-2, IFN-*γ*), Th2 (IL-4, IL-10), and Treg (Foxp3) were measured before and after the intervention at the middle luteal phase (6∼7 days after LH peak, progesterone ≥5 ng/ml). It is known that successful pregnancy is associated with the maternal immune, and we also extracted the ratio of Th2 and Th1 (Th2/Th1).

4 ml of complete blood was collected by vacuum blood collection vessel anticoagulant with heparin sodium. The peripheral blood mononuclear cells (PBMC) were isolated and cultured. The samples were stored in 4°C refrigerators and analyzed within 24 hours. Flow cytometry was used to detect the level of Treg cells (BD Cytofix/Cytoperm) in peripheral blood mononuclear cells using ™ fixation/permeabilization solution kit (554714, BD company, CA, USA), CD4-FITC (eBioscience, 11-0049-41), and CD25-PE (eBioscience, 12-0259-42). The level of IL-2, IFN-*γ*, IL-4, IL-10, and Foxp3 was detected in an enzyme-linked immunosorbent assay (ELISA). The specific steps were strictly followed as per the instructions mentioned in the kit. Additional details on the experimental instruments and equipment, sample collection steps, and cytokines detection steps are provided in Supplementary Materials 1.

### 2.9. Maternal and Child Health during the Perinatal Period

The pregnancy and perinatal outcomes were obtained by follow-up with the patient as per the Society of Assisted Reproductive Technologies (SARTs) reporting guidelines through systematic medical records inquiry, face-to-face consultation, telephone, or WeChat inquiry. Maternal health indicators included Down's syndrome screening, the incidence of pregnancy complications and pregnancy-specific diseases, and delivery and postpartum conditions to evaluate maternal health. The neonatal outcomes will be recorded, and the neonatal development will be observed by follow-up in 3 months and 1 year.

### 2.10. Statistical Analysis

To assess the effect of missing data, an intention-to-treat (ITT) analysis was planned and we performed post-hoc sensitivity analyses, fitting best- and worst-case scenarios. For the best-case scenario, we assumed all unknown events in the treatment group were positive (clinical pregnancy) and those in the control group were negative. For the worst-case scenario, we assumed none of the women with missing data in the treatment group became pregnant, and all of the women with missing data in the control group did become pregnant.

SPSS 25.0 statistical software was used for statistics, and the measurement data were expressed as $\bar{X}\pm s$. The data were normal distribution, the independent sample *t*-test was used for intergroup comparison, and paired sample *t*-test was used for intragroup comparison; if the data were skew distribution, the Mann–Whitney *U* test was used between groups, Wilcoxon signed rank-sum test, case/control association analysis, and chi-square test were used before and after the group, and the difference was statistically significant (*p* < 0.05).

## 3. Results

### 3.1. Patient Enrollment

**Figure 1:**
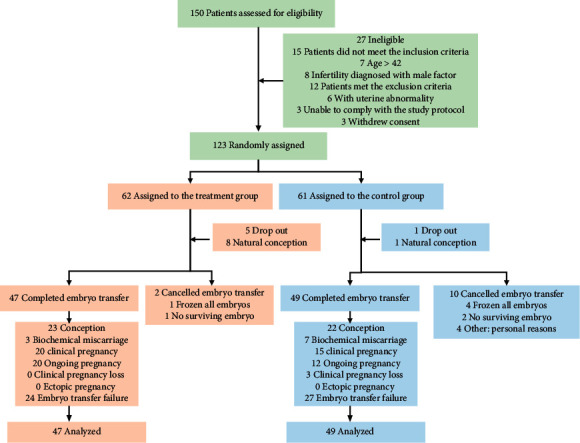
CONSORT 2010 flow diagram.

A total of 150 women were assessed for eligibility in the trial. However, fifteen patients did not meet the inclusion criteria and twelve patients met the exclusion criteria (Figure 1). Finally, 123 patients were randomly assigned to either the intervention group (62 cases) or the control group (61 cases). During the three-month intervention phase, 5 participants in the TG and 1 in the CG dropped out and 8 participants in the TG and 1 in the CG got natural conception. In the IVF-ET phase, 2 participants (1 was frozen all embryos and 1 was no surviving embryos) in the TG and 10 (4 were frozen all embryos, 2 were no surviving embryos, 4 were canceled for personal reasons) in the CG were canceled ET (Figure 1). Therefore, 108 women completed the three-month intervention phase (49 in the TG, 59 in the CG). Ninety-six women completed the follow-up (47 in the TG, 49 in the CG). Recruitment took place between March 2017 and February 2020. The TG and the CG were comparable in baseline demographics (Table 1). As for the outcomes of ovarian stimulation and embryo culture, the A-type endometrial pattern (EMP) on the hCG day was significantly higher in the TG (*p* = 0.033), and there were no significant differences between TG and CG (*p* > 0.05) regarding several mature ova, several D3 embryos, and stage of embryo transferred (Table 2). The mean (SD) age of the participants was 30.9 (4.02) years. 24.2% of women in the TG, and 23.0% in the CG were diagnosed with unexplained RIF.

### 3.2. The Pregnancy Outcomes after IVF-ET

As the primary outcome, CPR was significantly higher in the TG than in the CG (28 of 62 [45.2%] in the TG vs. 16 of 61 [26.2%] in the CG; relative risk: 1.72; 95% CI: 1.04–2.85; *p*=0.029) (Table 3). Among the second outcomes of pregnancy, natural pregnancy rate and ongoing pregnancy rate were also significantly higher in the TG than in the CG (Table 3). There was a trend suggesting that the CAM therapy may perform more successfully in avoiding biochemical miscarriage (RR: 0.318; 95% [CI]: 0.09–1.10, *p*=0.052). However, the rates of conception per woman, clinical pregnancy per IVF cycles, clinical pregnancy per ET cycles, and clinical pregnancy loss were similar between the two groups (Table 3).

For the post-hoc outcomes in clinical pregnancy, the best-case sensitivity analysis found significant improved differences in the TG than the CG (RR: 2.15; 95% CI: 1.34–3.46; *p* < 0.001), whereas the worst-case sensitivity analysis found no difference (RR: 1.02; 95% confidence interval (CI): 0.69–1.51; *p*=0.92) (Table 3). Moreover, we did not determine any side effects associated with CAM treatment.

### 3.3. The Expression Levels of Th1 (IL-2, IFN-*γ*), Th2 (IL-4, IL-10), and Treg (Foxp3) in Peripheral Blood

**Figure 2:**
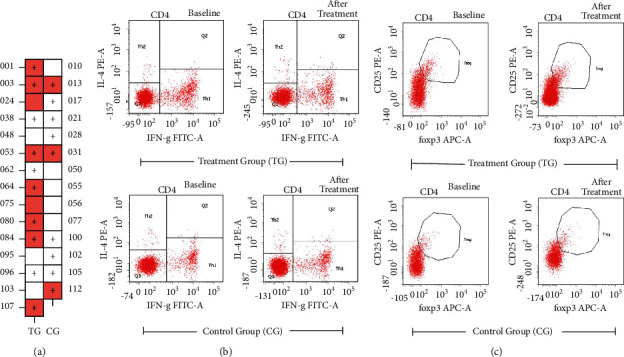
The differential expression analysis of unexplained RIF on Th1, Th2, and Treg between the TG and the CG. (a) The clinical pregnancy outcome between the TG and the CG. (“+” means successful clinical pregnancy, red square means agreed to the blood test. 9 of 15 [60.0%] in the TG vs. 3 of 14 [21.4%] in the CG; *p*=0.035); (b) Th1, Th2 flow cytometry profiles; (c) Treg flow cytometry profiles. The abscissa represents the fluorescence signal and the ordinate represents the number of cells.

For unexplained RIF, subgroup analysis was analyzed between the two groups, CPR was significantly higher in the TG than in the CG (9 of 15 [60.0%] in the TG vs. 3 of 14 [21.4%] in the CG; *p* = 0.035) (Figure 2(a)). Nineteen blood samples (9 from the TG and 10 from the CG) of unexplained RIF were collected from the two groups, respectively (5 patients in the TG and 4 patients in the CG refused blood sampling) for flow cytometry and the ELISA test. One sample in the TG for flow cytometry becomes hemolysis. When serum data were analyzed before and after the intervention, there were variations in different T lymphocyte expressions. Based on our observations on the flow cytometry outcome of PBMC, there were significant differences in Th2/Th1, and Treg expressions in the TG than in the CG after the intervention (i.e., Th2/Th1 13.18 ± 7.15 vs. 7.22 ± 3.52, TG vs. CG after the intervention, *p* < 0.05) (Table 4, Figures 2(b) and 2(c)). Moreover, based on the observation of ELISA, IL-2, IL-10, and Foxp3 were significantly higher in the TG than in the CG after the intervention (i.e., Foxp3 220.66 ± 82.2 vs. 208.66 ± 91.36, the TG vs. the CG, after the intervention, *p* < 0.05) (Table 5).

### 3.4. Maternal Child Health Outcomes during the Perinatal Period

The perinatal outcomes of 41 pregnant women (28 in the TG and 13 in the CG) and 56 neonatal outcomes were obtained by the follow-up (natural conceptions were included). There was no significant difference in the Down's syndrome-related screening, the pregnancy-related complications and idiopathic diseases, and delivery, postpartum condition, and related complications. And the basic characteristics of newborns, neonatal complications, and neonatal development in 3 months and 1 year were similar between the two study groups (*p* > 0.05) (Tables 6 and 7).

## 4. Discussion

In this single-center, randomized trial involving 123 patients diagnosed with RIF who underwent IVF-ET, our study demonstrates that CAM therapy (ZYF with auricular acupressure) is efficient and safe in improving the pregnancy outcomes of RIF women who is undergoing IVF-ET, including the clinical pregnancy rate and ongoing pregnancy rate. The balance of the immune system during embryo implantation is considered to be based on the change of T lymphocyte expression in unexplained RIF women. We believe this is the first time to evaluate the efficacy and maternal and child health of the CAM therapy (ZYF with auricular acupressure) for the RIF and a correlation between serum T lymphocyte expression and the use of the CAM therapy in IVF cycles has been reported.

About 1∼3% of women experience early recurrent implantation failure; about 50% of patients are associated with unexplained RIF [21–23]. Many studies have found that the cytokine balance of Th1/Th2/Th17/regulatory T cells (Treg) plays an important role in the maintenance of embryo implantation and normal pregnancy, especially for unexplained RIF [23]. The disorder of the cytokine balance will lead to repeated implantation failure and recurrent abortion. Th1 cells secrete a variety of cytokines, mainly interleukin (IL)-2, tumor necrosis factor (TNF)-*α*, and interferon (IFN)-*γ*, which mediate cellular immunity and participate in pro-inflammatory response. Th2 cells mainly secrete IL-4, IL-5, IL-6, IL-10, and IL-13; mediate humoral immunity; and participate in maternal and fetal immune tolerance and anti-inflammatory response. Treg cells mainly secrete IL-10, IL-35, and transforming growth factor (TGF)-*β*, which participate in maternal and fetal immune tolerance and maintain normal pregnancy. Th1/Th2/Th17/Treg cytokines can affect the maternal immune system, regulate the angiogenesis of the maternal-fetal interface, and play an important role in all stages of pregnancy [7].

**Figure 3:**
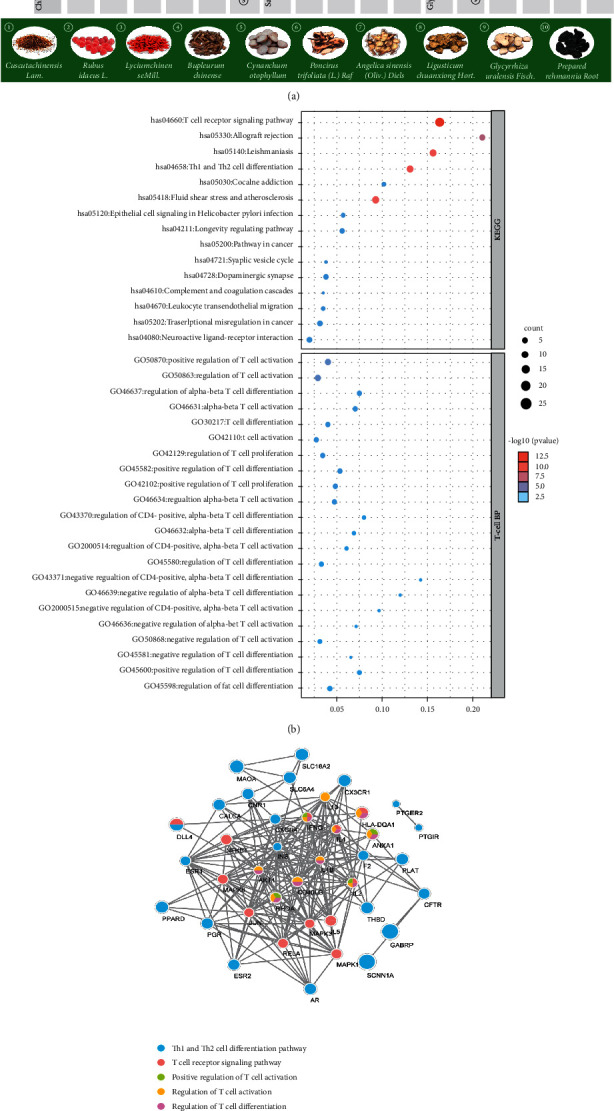
Previous work of the network pharmacology on the regulation of RIF by ZYF. (a) The core active ingredients of ZYF. The green rectangle displays the picture of each herb of the ZYF with a number label; the grey bars display the name of the core ingredients of each herb marked with the herb label. The upper box displays the gene target degree and the relative content of the core ingredients with RIF (higher degree means more relevant); (b) the bubble diagram exhibiting the pathways for the intersected target proteins. The vertical axis represents the pathway name, while the horizontal axis shows the enrichment gene number. The larger the ratio of enriched genes to the total genes, the larger the dot size is. (c) “Target-pathway” network diagram. The circles represent the signal pathway genes, while the colors of the circles represent the pathway name.

According to the TCM theory, RIF is associated with kidney essence deficiency, liver stagnation, and blood stasis [24, 25]. Therefore, improving the physical condition of the kidney, liver, and blood may improve the pregnancy outcomes of RIF. Significant efficacy has been demonstrated for subfertile women by Chinese herbs [25, 26] and acupuncture [12, 27]. The ZYF is composed of Tu Sizi (*Cuscuta chinensis Lam.*), Fu Penzi (*Rubus idaeus L.*), Gou Qizi (*Lyciumchinense Mill.*), Chai Hu (*Bupleurum chinense*, Bai Shao (*Cynanchum otophyllum*), Zhi Ke (*Poncirus trifoliata (L.) Raf*), Dang Gui (*Angelica sinensis (Oliv.) Diels*), Chuan Xiong (*Ligusticum chuanxiong Hort.*), Shu Dihuang (*Rehmannia glutinosa (Gaert.) Libosch. ex Fisch. et Mey.*), and Zhi Gancao (*Glycyrrhiza uralensis Fisch.*), which are classic herbs in nourishing the kidney, smoothing the liver, and circulating the blood to improve the CPR. Moreover, searching the HERB database of the herbs, ellagic acid, astragalin, acteoside, ferulic acid, and gallic acid were considered the most relative bioactive molecules for RIF (based on the gene target degree, Figure 3(a)), which are essential for the development of the embryo, such as astragalin and ferulic acid can regulate the balance the immune-related signaling way to suppress anti-inflammatory in uterine [28]. However, excessive amounts of ellagic acid (dose >1208 mg/kg b.w.) will induce abortion (Table 8).

Our findings of ZYF with auricular acupressure in regulating the process of immune tolerance during embryo transfer verified the network pharmacology analysis. Based on the network pharmacology of ZYF for RIF, KEGG pathway enrichment showed that the T-cell receptor pathway, Th1, and Th2 cell differentiation are prominent pathways (Figure 3(b), Table S6). Furthermore, IL-4, IL-10, HLA-DQA1, NF-*κ*B, and ANXA1 signal pathway genes are enriched in large numbers, which are the key pathways for ZYF to treat RIF, as shown in Figure 3(c). There is a close synergy between key pathways, which can regulate the key biological processes of maternal-fetus balance during the embryo implantation, especially for immune regulation and other related targets.

Pregnancy is a physiological process greatly dependent on immune tolerance. In our study, the results of a higher level of Th2/Th1 and Treg expression compared with the control group are consistent with the improvement of pregnancy outcomes. Furthermore, the secretion of IL-2 (produced by Th1) was decreased. IL-10 (produced by Th2) and Foxp3 (produced by Treg) were increased in the TG than in the CG. T lymphocytes play a critical role in regulating the immune response and maintaining immune tolerance both physiological and pathological during embryo implantation [53]. Recent studies have shown that pregnancy is a process of mutual conversion between pro-inflammatory and anti-inflammatory conditions [54, 55]. The status of pregnancy was divided into three distinct immunological stages: at the first beginning of ET, a pro-inflammatory stage is performed [56]; in the second status, for fetal growth, an anti-inflammatory-oriented stage is performed [57, 58]; and third, an anti-inflammatory status switched to a pro-inflammatory stage for the initiation of labor [59]. Some studies have demonstrated in RIF patients, a predominant Th2/Treg immunity (which produces anti-inflammatory cytokines) underlies normal pregnancy [54], whereas a predominant Th1 immunity (which produces pro-inflammatory cytokines) has been observed in women with recurrent miscarriage [60]. Accordingly, the balance of Th2/Th1 and the shift from pro-inflammatory to anti-inflammatory status has been linked to positive pregnancy outcomes in those undergoing IVF [61], which is also important for RIF in the early stage of ET.

In agreement with previous studies [10], we demonstrated no side effects of the CAM therapy in infertile women who underwent IVF-ET. In addition, our findings suggest that CAM therapy has no increased risk of maternal and child health in treating RIF who underwent IVF-ET. According to the Society of Assisted Reproductive Technologies (SARTs) reporting guidelines, the perinatal outcomes (Down syndrome and pregnancy or delivery-related complications and idiopathic diseases) and the neonatal outcomes (birth weight, Apgar score, and neonatal complications) were obtained by follow-up during the pregnancy, and three months and 1 year after born. Furthermore, the safety of the CAM therapy is demonstrated with durable effects 1 year after giving birth. To our best knowledge, several large epidemiological studies [62, 63] have concerned about the perinatal outcomes such as preterm delivery and low birth weight of the ART-conceived infants without inconsistent results. However, enough follow-up needs more observation, especially for CAM therapy.

## 5. Limitation

This trial has limitations. First, placebo ZYF and sham auricular acupressure should be administrated for potential placebo efficacy. Second, successful pregnancy is a complex process, and we explore the mechanism based on the network pharmacology only.

## 6. Conclusion

In conclusion, the CAM therapy (ZYF with auricular acupressure) has achieved the balance of the immune system during ET for RIF. The mechanism may be mediated by upregulating the expression of Treg and Th2/Th1 pattern, which can help embryo implantation. In addition, concerning the perinatal outcomes and the neonatal outcomes, the CAM therapy indicated the safety for the RIF women. For further study, we will screen the main active ingredients of ZYF in the treatment of RIF and combine metabolomics with proteomics to fuse various metabolic pathways and regulatory pathways to jointly elucidate the overall status of biological systems and construct the expression regulatory network of the final metabolites in the further study. Finally, the protocol of three months before IVF-ET might be a burden to patients in other countries.

## Figures and Tables

**Table 1:** Baseline characteristics of trial participants.

Characteristics, mean (SD)	TG (*n* = 62)	CG (*n* = 61)	*P*-value
Age (year)	30.90 ± 4.02	30.87 ± 4.26	0.91
BMI (kg/m^2^)	21.32 ± 2.66	21.66 ± 4.22	0.35
Infertility history (year)	4.47 ± 2.99	4.70 ± 2.91	0.64

The baseline of ovarian function, mean (SD)			
FSH (IU/L)	6.39 ± 1.13	6.43 ± 1.10	0.90
LH (IU/L))	5.05 ± 1.25	4.81 ± 1.34	0.31
Estradiol (pmol/L)	47.22 ± 13.52	45.35 ± 13.75	0.42
AFC in both ovaries	15.19 ± 3.92	15.43 ± 3.93	0.74

Infertility diagnosis, *n* (%)			
Anovulation	4/62 (6.5)	4/61 (6.6)	0.63
Endometriosis	5/62 (8.2)	5/61 (8.2)	0.62
Tubal	4/62 (6.5)	4/61 (6.6)	0.63
Immunologic derangement	5/62 (8.1)	6/61 (9.8)	0.49
Unexplained	15/62 (24.2)	14/61 (23.0)	0.52
More than two factors	29/62 (46.8)	28/61 (45.9)	0.53

Values are mean ± SD or *n* (%). There were no significant differences between groups (*P* > 0.05) in any of the baseline characteristics. FSH = follicle-stimulating hormone. LH = luteinizing hormone. AFC = Antral follicle count.

**Table 2:** Outcomes of ovarian stimulation and embryo transfer.

Items	TG (*n* = 49)	CG (*n* = 59)	*P*-value
No. of mature ovum retrieved	13.38 ± 4.11	12.15 ± 4.01	0.12

No. of D3 embryos	6.27 ± 2.08	6.19 ± 2.45	0.85

Stage of embryo transferred			
Blastocyst transfer^*∗*^	38/47 (80.9)	39/49 (79.6)	0.88
Cleavage-stage embryo transfer^*∗*^	9/47 (19.2)	10/49 (20.4)	

Endometrial parameters (on hCG day)			
Endometrial thickness	10.60 ± 2.05	10.14 ± 2.05	0.25
Endometrial pattern (A%)	30/49 (61.2)	24/59 (40.7)	**0.03**

^*∗*^Two patients in the TG were canceled for embryo transfer (1 for frozen all embryos and 1 for no surviving embryo); 10 in the CG were canceled for embryo transfer (4 for frozen all embryos, 2 for no surviving embryos, and 4 for other reasons). Values are mean ± SD or *n*(%).

**Table 3:** Comparison of the pregnancy outcomes^*∗*^.

Items	TG (*n* = 62)	CG (*n* = 61)	Relative risk in TG vs. CG (95% CI)	*p*-value
The primary outcome				
Clinical pregnancy rate	28/62 (45.2)	16/61 (26.2)	1.72 (1.04–2.85)	**0.02**

The second outcomes				
Natural pregnancy rate	8/62 (12.9)	1/61 (1.6)	7.78 (1.05–61.06)	**0.04**
Conception rate	31/62 (50.0)	23/61 (37.7)	1.33 (0.88–1.99)	0.17
Clinical pregnancy per IVF cycles	20/49 (40.8)	15/59 (25.4)	1.61 (0.93–2.79)	0.08
Clinical pregnancy per ET cycles	20/47 (42.6)	15/49 (30.6)	1.39 (0.81–2.38)	0.22
Ongoing pregnancy rate	28/47 (59.6)	13/49 (26.5)	1.60 (1.08–2.33)	**0.004**

Live birth rate	**28/47 (59.6)**	**13/49 (26.5)**	**0.24 (0.10–0.58)**	**0.001**
Singleton	23/47 (48.9)	12/49 (24.5)	2.96 (1.24–7.03)	**0.01**
Twin	4/47 (8.5)	1/49 (2.0)	4.47 (0.48–41.51)	0.20
Triplet	1/47 (2.1)	0/49 (0)	—	0.49

Pregnancy loss				
Biochemical miscarriage rate	3/31 (9.7)	7/23 (30.4)	0.318 (0.09–1.10)	**0.05**
The clinical pregnancy loss rate	0/31 (0.0)	3/23 (13.0)	—	0.14
Ectopic pregnancy rate	0/31 (0.0)	0/23 (0.0)	—	—

Post-hoc sensitivity analysis				
Best case for TCM¶	35/62 (56.5)	16/61 (26.2)	2.15 (1.34–3.46)	**<0.001**
Worst case for TCM§	28/62 (45.2)	27/61 (44.3)	1.02 (0.69–1.51)	0.92

^*∗*^Natural conception was calculated. ¶Assuming all unknown events in the treatment group were positive, and those in the control group were negative. § Assuming none of the women with missing data in the treatment group became pregnant, and all of the women with missing data in the control group did become pregnant. Values are mean ± SD or *n*(%). The definition and calculation of indicators are shown in Table S7.

**Table 4:** Serum of T lymphocyte by flow cytometry.

Items	Treatment group (*n* = 9)		Control group (*n* = 10)	
	Baseline	After intervention	Baseline	After intervention
Th1 expression, % parent	14.62 ± 7.48^**‡**^	11.83 ± 2.28	21.15 ± 9.83	16.79 ± 7.85^**‡**^
Th2 expression, % parent	1.40 ± 1.11^**‡**^	1.53 ± 0.95^**‡**^	1.23 ± 0.70 ^**‡**^	1.21 ± 0.68^**‡**^
Th2/Th1 expression, % parent	9.23 ± 6.05^**‡**^	13.18 ± 7.15^*∗*#^	6.07 ± 3.11	7.22 ± 3.52
Treg expression, % parent	5.46 ± 1.27	7.87 ± 1.75^*∗*#^	5.4 ± 1.83	5.89 ± 1.54

‡The data showed a skewed distribution, the Mann-Whitney*U* test was used for comparison between groups, and the Wilcoxon signed-rank test was used for comparison before and after treatment within groups; ^*∗*^*p* < 0.05, TG vs. CG (after intervention); ^#^*p* < 0.05, ^##^*p* < 0.01, TG (after intervention) vs. TG (baseline). Values are mean ± SD.

**Table 5:** T lymphocyte-related factor by ELISA.

Items	Treatment group (*n* = 10)		Control group (*n* = 10)	
	Baseline	After intervention	Baseline	After intervention
IL-2 (Th1 secreted factor), pg/ml	208.15 ± 55.34	151.24 ± 52.58^*∗*#^	187.07 ± 50.41	192.61 ± 61.97
IFN-*γ* (Th1 secreted factor), pg/ml	249.00 ± 59.74	177.93 ± 65.57^##^	240.19 ± 44.13^**‡**^	264.09 ± 54.45
IL-4 (Th2 secreted factor), pg/ml	17.24 ± 8.81	27.13 ± 10.99^##^	18.52 ± 14.25	19.97 ± 11.83^**‡**^
IL-10 (Th2 secreted factor), pg/ml	37.11 ± 15.13	61.66 ± 21.31 ^*∗*##^	32.99 ± 14.20	33.5 ± 11.20
Foxp3 (Treg regulatory factor), pg/ml	192.05 ± 89.74^**‡**^	220.66 ± 82.2^**‡**^^*∗*#^	196.99 ± 67.08	208.66 ± 91.36^**‡**^

^**‡**^The data showed a skewed distribution, the Mann–Whitney *U* test was used for comparison between groups, and the Wilcoxon signed-rank test was used for comparison before and after treatment within groups; ^*∗*^*p* < 0.05, TG vs. CG (after intervention); ^#^*p* < 0.05, ^##^*p* < 0.01, TG vs. TG (baseline); values are mean ± SD.

**Table 6:** Perinatal outcomes.

Items	TG (*n* = 28)	CG (*n* = 13)	Relative risk in TG vs. CG (95% CI)	*p*-value
Down's syndrome-related screening				
Nuchal Translucency (NT)	1/28 (3.6)	0/13 (0.0)	**—**	0.49
Oscar test	0/17 (0.0)	0/9 (0.0)	**—**	**—**
Noninvasive DNA examination	0/14 (0.0)	0/11 (0.0)	**—**	**—**
Amniocentesis	0/3 (0.0)	0/1 (0.0)	**—**	**—**

Pregnancy-related complications and idiopathic diseases				
Placenta previa	1/28 (3.6)	0/13 (0.0)	—	0.49
Oligohydramnios	1/28 (3.6)	1/13 (7.7)	0.44 (0.26–7.71)	0.54
Premature rupture of membranes (PROM)	3/28 (10.7)	2/13 (15.4)	0.66 (0.96–4.52)	0.64
Gestational diabetes	3/28 (10.7)	2/13 (15.4)	0.66 (0.96–4.52)	0.64
Gestational hypertension	2/28 (7.1)	1/13 (7.7)	0.92 (0.76–11.20)	1.00
Intrahepatic cholestasis of pregnancy (ICP)	2/28 (7.1)	1/13 (7.7)	0.92 (0.76–11.20)	1.00
Pregnancy anemia	1/28 (3.6)	2/13 (15.4)	0.92 (0.76–11.20)	0.23
Thrombocytopenia during pregnancy	1/28 (3.6)	0/13 (0.0)	—	1.00
Pregnancy with thyroid disease	2/28 (7.1)	1/13 (7.7)	0.92 (0.76–11.20)	1.00

Delivery, postpartum condition, and related complications				
Gestational week of delivery, week	37.50 ± 1.09	37.00 ± 1.08	—	
Vaginal delivery, *n* (%)	6/28 (21.4)	2/13 (15.4)	1.50 (0.26–8.69)	1.00
Cesarean section, *n* (%)	22/28 (78.6)	11/13 (85.6)	0.67 (0.12–3.86)	1.00
Adherent placenta, *n* %)	1/28 (3.6)	0/13 (0.0)	—	1.00
Abnormal lochia, *n* (%)	6/28 (21.4)	3/13 (23.1)	0.91 (0.19–4.39)	1.00
Oligogalactia, *n* (%)	4/28 (14.3)	2/13 (15.4)	0.92 (0.15–5.78)	1.00

Values are mean ± SD or *n* (%).

**Table 7:** Neonatal outcomes.

Items	TG		CG	
	Boys (*n* = 19)	Girls (*n* = 15)	Boys (*n* = 10)	Girls (*n* = 4)
Basic characteristics of newborn				
Birth weight, kg	3.23 ± 0.21	3.16 ± 0.34	3.21 ± 0.24	3.06 ± 0.28
Birth height, cm	50.68 ± 3.40	49.63 ± 3.17	49.30 ± 3.44	48.25 ± 4.44
Birth deformity, no. (%)	0/19 (0.0)	0/15 (0.0)	0/10 (0)	1/4 (25.0)
Apgar score ＜7	0/19 (0.0)	1/15 (6.7)	0/10 (0)	0/4 (0)

Neonatal complications				
Jaundice of the newborn, no. (%)	2/19 (10.5)	2/15 (13.3)	2/10(20.0)	1/4(25.0)
Pneumonia of the newborn, no. (%)	1/19 (5.3)	1/15 (6.7)	0/10 (0)	0/4 (0)
Hypoglycemia of the newborn, no. (%)	2/19 (10.5)	1/15 (6.7)	1/10(10.00)	0/4 (0)

Neonatal development				
Weight after 3 months, kg	5.93 ± 0.69	6.03 ± 0.62	6.23 ± 0.50	5.83 ± 0.47
Weight after 1 year, kg	9.91 ± 1.80	9.35 ± 1.13	9.70 ± 0.71	9.20 ± 0.83
Height after 3 months, cm	60.74 ± 2.63	61.07 ± 2.24	61.30 ± 2.24	61.50 ± 0.50
Height after 1 year, cm	78.53 ± 4.30	76.73 ± 4.63	75.50 ± 3.26	77.25 ± 5.85
History of pneumonia, no. (%)	1/19 (5.3)	1/15 (0.0)	1/10 (10.0)	0/4 (0.0)
History of diarrhea, no. (%)	1/19 (5.3)	0/15 (0.0)	2/10 (20.0)	0/4 (0.0)
History of other diseases, no. (%)	2/19 (10.5) **‡**	0/15 (0.0)	0/10 (0.0)	1/4 (25.0)¶

Follow-up for 1 year for perinatal outcomes in successfully delivered infants, data are *n*(%), mean (SD), or *n*/*N*(%). ‡One person died unexpectedly, and the other one suffered from renal failure; ¶diagnosed with congenital heart disease.

**Table 8:** Toxicological and pregnancy-related pharmacological reports of core ZYF active ingredients.

ZYF core ingredients	Report on toxicity-relatedeffects of ZYF ingredients^*∗*^	Frontier report on the pharmacological effects of ZYF on pregnancy
Chlorogenic acid	Intraperitoneal injection (ip) of over 4 g/kg b.w. may cause death in rats; injection of over 40 mg/kg 5–12 days after conception may lead to abnormal development of the fetal musculoskeletal system	Chlorogenic acid (50 *μ*mol/L) supplementation can improve the maturation, fertilization, and development of oocytes, and improve the quality of embryonic development and electroporation-treated embryos [29]

Ellagic acid	Injection of excessive amounts of ellagic acid (dose >1208 mg/kg b.w.) induced abortion after 16 days of gestation in females, with specific developmental abnormalities in the blood and lymphatic systems	Ellagic acid (10 *μ*M) protects zebrafish embryonic development from oxidative DNA damage, improves embryo survival, and improves morphological parameters in larvae *in vitro* [30]; ellagic acid (60 mg/kg) shows anti-senescence protection in rat embryonic fibroblasts [31]

Hyperoside	No relevant toxicological reports have been published	Hyperoside (40 mg/kg b.w.) can reduce pregnancy loss in anticardiolipin antibody-positive rats by regulating the mechanistic target of rapamycin (mTOR)/S6K and Toll-like receptor (TLR)-4/MyD88/nuclear factor-kappa B(NF-*κ*B) signaling pathways [32].

Isoquercitrin	Details of toxic effects have not been reported other than a lethal dose value (rat, ip >5 g/kg b.w.)	Isoquercitrin (100 mg/kg b.w.) alleviates nitrosation, oxidation, and endoplasmic reticulum stress by inhibiting nitric oxide synthase (NOS)-2 and increasing the expression of superoxide dismutase (SD)-1, to treat neural tube defects in embryos of diabetic mothers [33]

Acteoside	Details of toxic effects did not report other than a lethal dose value (rat, oral (po) >5 g/kg b.w.)^*∗*^	The addition of acteoside (10～50 *μ*M) during *in vitro* maturation (IVM) increases the blastocyst formation rate and significantly reduces reactive oxygen species (ROS). Furthermore, in acteoside-treated oocytes, cytoplasmic maturation is improved and mitochondria and lipid droplets are evenly distributed throughout the cytoplasm [34]

Astragalin	No relevant toxicological reports have been published	Astragalin (100 *μ*g/mL) over regulates the NF-*κ*B/mitogen-activated protein kinase (MAPK) signaling pathway to suppress inflammatory responses in murine *Leptospira*-infected uterine and endometrial epithelial cells [35]

Kaempferol	Kaempferol at a dose of 25 mg/l can induce mutations in mammalian (hamster ovary) somatic cells	Kaempferol (0.1 *μ*M) improves embryonic development by inhibiting oxidative stress in porcine oocytes and reducing apoptosis [36]; kaempferol (0.05～1 *μ*M) reduces mitochondrial dysfunction and abnormal oxidative stress induced by H_2_O_2_ during embryonic development [37]

Saikosaponin D	Details of toxic effects were not reported other than a lethal dose value (ip > 1530 mg/kg b.w.)	Saikosaponin D (5～50 mg/kg b.w.) has a weak estrogen-like effect *in vivo* and may be a potential plant estrogen [38]; its regulatory effect on reproductive diseases is not clear

Paeoniflorin	Excessive intake of paeoniflorin can lead to sleep behaviors in mice (ip > 3530 mg/kg b.w.)	Paeoniflorin (8 mg/kg b.w.) can enhance endometrial receptivity of luteal insufficiency (RU486-induced model) mice through the leukemia inhibitory factor [39]; Paeoniflorin (60 mg/kg b.w.) can also improve depression-like behavior in offspring of prenatally stressed mothers by restoring hypothalamic–pituitary–adrenal (H–P-A) axis and glucocorticoid receptor related dysfunction [40]

Naringin	Excessive naringin use in guinea pigs is lethal when naringin administration reaches 1250 mg/kg b.w. per day; rats experience weight loss, etc. Naringin overdose in guinea pigs is lethal (ip >2 g/kg b.w.)	The bi-directional regulatory functions of naringin's estrogenic and anti-estrogenic activities may present deleterious disturbances to endocrine regulation in women. However, it was reported that naringin does reduce fertility or cause early embryonic developmental toxicity even when the intervention dose reached 1250 mg/kg b.w. in rats [41]

Hesperidin	Details of toxic effects are not reported other than a lethal dose value (ip > 1 g/kg b.w.)	A potential preventive effect of hesperidin (50 mg/kg b.w.) on formaldehyde (2 mg/kg b.w.) toxicity was found in pregnant rats [42]; the use of hesperidin (1 mg/kg b.w.) during pregnancy has a positive effect on the reflex motor behavior of mouse offspring, which may stem from its antioxidant activity [43]

Liquiritin	No relevant toxicological reports have been published	Liquiritin and glycyrrhizic acid (0.2～0.4 mg/mL) can resist uterine contractile spasms caused by oxytocin by inhibiting the phosphorylation of heat shock protein (HSP)-27 [44]

Glycyrrhizic acid	Glycyrrhizic acid doses >662 mg/kg b.w. may cause seizures in humans. The lethal dose in mice (po) is >3 g/kg b.w.	

Ferulic acid	IP injection of 350 mg/kg ferulic acid can cause ataxic behaviors such as rigidity in mice; an intravenous dose >857 mg/kg b.w. resulted in mouse death	Ferulic (40～120 *μ*M) acid inhibits NK-*κ*B and MAPK pathways and inhibits lipopolysaccharide-induced inflammation in endometrial epithelial cells [28]; ferulic acid (20 mg/kg b.w.) protects pancreatic islets in pregnant diabetes rats insulin *β* cell [45]. It can also improve placental inflammation and pathological apoptosis in preeclampsia rats [46]

Amygdalin	An oral dose of amygdalin >50 mg/kg b.w. causes nausea or vomiting in human infants and even gastrointestinal dyspnea; ip injection dose >167 mg/kg b.w. will lead to drowsiness or significant weight change in primates; ip injection >405 mg/kg b.w. causes mouse death	Treatment of endometriosis rats with a combination of atorvastatin and amygdalin (5 mg/kg b.w.) modulates the expression of TNF-*α*, IL-6, matrix metalloproteinases (MMP)-2 and MMP-9 [47]; amygdalin is particularly important for female reproduction by modulating extracellular and intracellular signaling pathways involved in secretory activity, cell viability, steroidogenesis, proliferation, and apoptosis [48]

Catalpol	Oral catalpol administration >10 g/kg b.w. or intravenous injection (iv) >2500 mg/kg b.w. lead to mouse death	Catalpol (10～100 mg/kg b.w.) can inhibit the transmission of TLR4 signals, prevent the expression of its downstream NF-*κ*B/MAPK signal pathway, and significantly reduce the inflammatory cytokines IL-1*β*, IL-6, TNF-*α*, and chemokines C-X-C motif chemokine ligand (CXCL)-8 and CXCL-5, as well as myeloperoxidase (MPO) activity of uterine tissue, to implement anti-inflammatory and protective effects on endometrial tissue [49]

Gallic acid	Gallic acid, at oral doses >5 g/kg b.w., causes chronic pulmonary edematous liver in rabbits; subcutaneous injection at >5 g/kg b.w., iv >320 mg/kg b.w., or >4300 mg/kg b.w. ip is lethal in mice	Subcutaneous injection of gallic acid (dose >5 mg/kg b.w.) 1 day before the mating of female animals can affect ovary and fallopian tube function; gallic acid can inhibit the production of pro-inflammatory and labor-promoting mediators related to myometrial contraction and rupture of fetal membranes. Future preclinical studies may clarify the efficacy of gallic acid in the prevention of inflammatory preterm birth [50]

Ligustrazine	Ligustrazine oral administration >1910 mg/kg b.w., ip >800 *μ*g/kg b.w., or iv > 239 mg/kg b.w. will lead to mouse death	Ligustrazine (100 mg/kg b.w.) can delay the development and fibrosis of endometriosis mice by reducing the pathological aggregation of platelets and the pathological expression of epithelial-mesenchymal transition, fibroblast-to-myofibroblast transdifferentiation, and fibrogenesis markers [51]; ligustrazine (60 mg/kg b.w.) induction reduces preeclampsia by inhibiting trophoblast autophagy and promoting its survival and migration by regulating the miR-16-5p/IGF-2 axis [52]

Note: toxicological information with ^*∗*^ was obtained from the PubChem database (https://pubchem.ncbi.nlm.nih.gov/) and the CHEMSRC database (https://www.chemsrc.com/).

## Data Availability

All data used to support the finding of the study can be obtained from the corresponding author upon reasonable request.

## Abbreviations

RIF: Recurrent implantation failure
CPR: Clinical pregnancy rate
ART: Assisted reproductive technology
IVF-ET: In vitro fertilization and embryo transfer
COS: Controlled ovarian stimulation
TCM: Chinese traditional medicine
ZYF: Zhuyun formula
ET: Embryo transfer.

## Supplementary Materials

Supplementary Materials Trial Protocol (Supplementary material 1). CONSORT 2010 Checklist (Supplementary material 2).
