# Supplementary material for: The Efficacy and Safety of the Zhuyun Formula and Auricular Acupressure for the Infertile Women with Recurrent Implantation Failure: A Randomized Controlled Trial
Source: Evid Based Complement Alternat Med. 2022 Oct 11;2022:5274638. doi: 10.1155/2022/5274638 (PMC9578856; doi:10.1155/2022/5274638)
Supplement: Supplementary Materials — Trial Protocol (Supplementary material 1). CONSORT 2010 Checklist (Supplementary material 2). [file 5274638.f1.zip › 5274638.f1/Supplementary material 1-study protocol (1).docx]

**-****Trial Protocol-**

# The Efficacy and Safety of the Zhuyun Formula and Auricular Acupressure for the Infertile Women with Recurrent Implantation Failure: A Randomized Controlled Trial

# INTRODUCTION

## Background

Over the last 30 years, the clinical treatment of infertility has changed dramatically. Represented by *in vitro* fertilization and embryo transfer (IVF-ET), the rise of assisted reproductive technology (ART), has greatly improved the live birth rate (LBR) of infertile couples. However, LBR remains 36.2%[[8](#_ENREF_1)]. Recurrent implantation failure (RIF) is intractable infertility clinically and poses a global therapeutic challenge. RIF refers to cases in which women have had more than three failed IVF attempts with more than 4 good quality embryos[[1](#_ENREF_2)]. Around 5% of women are expected to suffer from two consecutive pregnancy losses, almost 75% are due to an implantation failure, and therefore are never recognized as clinical pregnancies[[04](#_ENREF_3)]. Thus, new therapies that will improve reproductive outcomes are highly desirable[[19](#_ENREF_4)].

### The use of ZYF with auricular acupressure by women undergoing IVF

Traditional Chinese Medicine (TCM), an ancient therapy, with a long history in treating infertility has shown promise in women with RIF by improving embryo development and LBR. Kidney deficiency, liver stagnation, and blood stasis in TCM play a universal role in prolonging the human reproductive period. TCM therapy is one of the most crucial parts of complementary and alternative medicine (CAM), which included Chinese herbs, acupuncture, tuina, taichi (exercise) et al. The Zhuyun Formula (ZYF) is composed of three classic prescriptions (Wuzi Yanzong Wan, Sini San, Siwu Decoction) which affect infertility by kidney-tonifying, liver-regulation, and blood-circulation. The formula included Tu Sizi (*Cuscuta chinensis Lam.*) 15g, Fu Penzi (*Rubus idaeus L.)*, Gou Qizi (*Lyciumchinense Mill.)* 10g, Chai Hu (*Bupleurum chinense*) 10g, Bai Shao (*Cynanchum otophyllum*) 15g, Zhi Ke (*Poncirus trifoliata (L.) Raf*) 10g, Dang Gui (*Angelica sinensis (Oliv.) Diels*) 10g, Chuan Xiong (*Ligusticum chuanxiong Hort.*) 10g, Shu Dihuang (*Rehmannia glutinosa (Gaert.) Libosch. ex Fisch. et Mey.*) 10g, Zhi Gancao (*Glycyrrhiza uralensis Fisch.*) (see **Table S1)[**[**08**](#_ENREF_7)**,**[**12**](#_ENREF_8)**,**[**04**](#_ENREF_9)**]**. Our previous study suggested that the CAM therapy (ZYF with auricular acupuncture) could increase the clinical pregnancy rate (CPR) of IVF-ET (CPR: 41.2% vs. 26.7%, treatment group vs. control group, *P* < 0.05), reduce the early abortion rate and adverse effects. However, a prospective, randomized clinical trial with a larger sample size should be conducted to further verify the efficacy and safety of the Chinese herbal formula in treating RIF, and especially for the mechanism of the detailed efficacy and safety of this herbal formula need to be further studied.

**Table S1** Composition and source of Chinese herbs of Zhuyun Formula prescription

| **Product Name** | **Scientific Name** | **Production unit** | B.N. | **place of production** | **Weight** |
| --- | --- | --- | --- | --- | --- |
| **TuSizi** | *Cuscuta chinensis Lam.* | Sichuan Guoqiang Herbal Pieces Co., Ltd | 19030104 | SiChuan | 15g |
| **FuPenzi** | *Rubus idaeus L.* | Sichuan BaiCaoYuan Pharmaceutical Co., Ltd | 190401 | ZheJiang | 10g |
| **GouQizi** | *LyciumchinenseMill.* | Sichuan Qianyuan Herbal Pieces Co., Ltd | 191201 | NingXia | 10g |
| **ChaiHu** | *Bupleurum chinense* | Sichuan Heyi nature medicine LTD. | 19090204 | Sichuan | 10g |
| **BaiShao** | *Cynanchum otophyllum* | Sichuan hengkangyuan Pharmaceutical Co., LTD. | 190801 | Anhui | 15g |
| **ZhiKe** | *Poncirus trifoliata (L.) Raf* | Chengdu jiankang pharmaceutical co.LTD | 191201 | Sichuan | 10g |
| **DangGui** | *Angelica sinensis (Oliv.) Diels* | Sichuan jinlin phaermaceutical co.LTD | 191201 | Gansu | 10g |
| **ChuanXiong** | *Ligusticum chuanxiong Hort.* | Sichuan hengkangyuan Pharmaceutical Co., LTD. | 190902 | Sichuan | 10g |
| **ShuDihuang** | *Rehmannia glutinosa (Gaert.) Libosch. ex Fisch. et Mey.* | Sichuan Guoqiang Herbal Pieces Co., Ltd | 19080105 | Henan | 10g |
| **ZhiGancao** | *Glycyrrhiza uralensis Fisch.* | Sichuan Herbal Pieces Co., Ltd | 191125 | Xinjiang | 5g |

###

### Network pharmacology of ZYF for RIF

With medical progressions, some studies have checked the significant efficacy of TCM, especially herbs and herbal compounds, in treating infertility of IVF. To identify the therapeutic mechanism of ZYF on specific target proteins involved in RIF, we performed network pharmacology of ZYF in the RIF target protein and biological regulation process.

We retrieved the core ingredients of TCMs by searching the HERB database (http://herb.ac.cn/), Traditional Chinese Medicine Systems Pharmacology Database and Analysis Platform (TCMSP) database (https://old.tcmsp-e.com/tcmsp.php), Traditional Chinese Medicine Integrated Database (TCMID) database (http://119.3.41.228:8000/tcmid), The Encyclopedia of Traditional Chinese Medicine(ETCM) database (http://www.tcmip.cn/ETCM/index.php/) in our previous period, then combined our previous High performance liquid chromatography-tandem mass spectrometry (HPLC/MS) identification results of ZYF and the ingredient data of published literature, and finally established core active ingredients based on the principle of Quality marker(Q-marker) theory 5 principles(Quality transmission and traceability, uniqueness, effectiveness, measurability and compatibility environment of ingredients) of TCMs. Use HERB (Related High-throughput Experiments) database, Swiss target prediction (Prediction probability > 0%) database (http://www. swisstargetprediction.ch/) and BATMAN (SCORE > 20) database (http://bionet.ncpsb. org/batman-tcm/) to predict the target network of core components of traditional Chinese medicine; CORE genes (DEGs) of RIF were analyzed on the Gene-Cards (https://www.genecards.org/) and Comparative Toxicogenomics Database (CTD) (http://ctdbase.org/). The interaction between RIF and the ZYF-predicted target proteins was identified by the Venn diagram. Enrichment analysis was conducted for the intersected target proteins by the Cytoscape software and meta-scape (https://metascape.org/). Additionally, the protein-protein interaction (PPI) network, biological processes, and KEGG signaling pathways were generated using the Cytoscape software (screening conditions: Min Overlap:3; P Value Cutoff:0.01; Min Enrichment:1.5).

We established 18 ingredients (including chlorogenic acid, elliptic acid, Hyperoside, Isoquercitrin, acteoside, astragalin, kaempferol, etc) according to the above method and Q-marker theory (see **Table S2** and **Figure S1**), and then we found out that the target genes of ZYF were 372 and those of RIF were 470. After the intersection, 51 target genes of drug-disease intersection were revealed (see **Table S3**). Additionally, the biological regulation processes of the ZYF-RIF intersection target genes include response to *lipopolysaccharide*, *regulation of inflammatory response*, and *inflammatory response* et al (see **Table S4**). The processes are related to immune tolerance during embryo implantation. To our best knowledge, during pregnancy, various immune effectors and molecules participating in the immune microenvironment establish specific maternal tolerance toward the semi-allogeneic fetus. Activated maternal immune effectors by the trophoblast antigens, such as T helper (Th), T cytotoxic (Tc), T regulatory (Treg), and B cells, are involved in the regulation of adaptive immunity. Moreover, we conducted an associated list of ZYF-RIF intersection target protein gene action T cell by Gene Ontology (go) analysis. As shown in **Table S5**, the network of ZYF intersection target genes with RIF mainly involved signal pathways such as *positive regulation of T cell activation and regulation of T cell activation*. String software was used to import into Cytoscape to further construct the key gene protein network mutual mapping of the core signaling pathway (see **Table S6**). Enrichment analysis results showed that *T cell receptor signaling pathway*, *allograft rejection*, *Th1, and Th2 cell differentiation and fluid shear stress, and atherosclerosis* are in higher degrees.

Thus, the abovementioned genes and biological regulation process of immune tolerance during embryo implantation might be involved in the pharmacological activity of ZYF in RIF and the T cell receptor signaling pathway, especially.

**Table S2** List of core active ingredients of ZYF

| **Ingredients** | **URL** | **Chem ID** | **Molecular Formula** | **Lipinskirules** | | | **OB**  **%** |
| --- | --- | --- | --- | --- | --- | --- | --- |
|  |  |  |  | **MV**  **(g/mol)** | **XLogP3-AA** | **HBD** |  |
| Chlorogenic acid | <https://pubchem.ncbi.nlm.nih.gov/compound/1794427> | 1794427 | C_16_H_18_O_9_ | 354.31 | -0.4 | 6 | 11.93 |
| Ellagic acid | <https://pubchem.ncbi.nlm.nih.gov/compound/5281855> | 5281855 | C14H6O8 | 302.19 | 1.1 | 4 | 43.06 |
| Hyperoside | <https://pubchem.ncbi.nlm.nih.gov/compound/5281643> | 5281643 | C21H20O12 | 464.40 | 0.4 | 8 | 6.94 |
| Isoquercitrin | <https://pubchem.ncbi.nlm.nih.gov/compound/5280804> | 5280804 | C21H20O12 | 464.4 | 0.4 | 8 | 1.86 |
| Acteoside | <https://pubchem.ncbi.nlm.nih.gov/compound/5281800> | 5281800 | C29H36O15 | 624.6 | -0.5 | 9 | 2.94 |
| Astragalin | <https://pubchem.ncbi.nlm.nih.gov/compound/5282102> | 5282102 | C21H20O11 | 448.4 | 0.7 | 7 | 14.03 |
| kaempferol | <https://pubchem.ncbi.nlm.nih.gov/compound/5280863> | 5280863 | C15H10O6 | 286.24 | 1.9 | 4 | 41.88 |
| Saikosaponin D | <https://pubchem.ncbi.nlm.nih.gov/compound/107793> | 107793 | C42H68O13 | 781.0 | 2.5 | 8 | 34.39 |
| Paeoniflorin | <https://pubchem.ncbi.nlm.nih.gov/compound/442534> | 442534 | C23H28O11 | 480.5 | -1 | 5 | 53.87 |
| Naringin | <https://pubchem.ncbi.nlm.nih.gov/compound/442428> | 442428 | C27H32O14 | 580.5 | -0.5 | 8 | 6.92 |
| Hesperidin | <https://pubchem.ncbi.nlm.nih.gov/compound/10621> | 10621 | C28H34O15 | 610.6 | -1.1 | 8 | 13.33 |
| Liquiritin | <https://pubchem.ncbi.nlm.nih.gov/compound/503737> | 503737 | C21H22O9 | 418.4 | 0.4 | 5 | 29.23 |
| Glycyrrhizic acid | <https://pubchem.ncbi.nlm.nih.gov/compound/14982> | 14982 | C42H62O16 | 822.9 | 3.7 | 8 | 19.62 |
| Ferulic acid | <https://pubchem.ncbi.nlm.nih.gov/compound/445858> | 445858 | C10H10O4 | 194.18 | 1.5 | 2 | 39.56 |
| Amygdalin | <https://pubchem.ncbi.nlm.nih.gov/compound/656516> | 656516 | C_20_H_27_NO_11_ | 457.4 | -2.7 | 7 | 4.42 |
| Catalpol | <https://pubchem.ncbi.nlm.nih.gov/compound/91520> | 91520 | C15H22O10 | 362.33 | -3.2 | 6 | 5.07 |
| Gallic acid | <https://pubchem.ncbi.nlm.nih.gov/compound/370> | 370 | C7H6O5 | 170.12 | 0.7 | 4 | 31.69 |
| ligustrazine | <https://pubchem.ncbi.nlm.nih.gov/compound/14296> | 14296 | C_8_H_12_N_2_ | 136.19 | 1.3 | 0 | 20.01 |

**Figure S1** Molecular structure of core active ingredients in ZYF

**
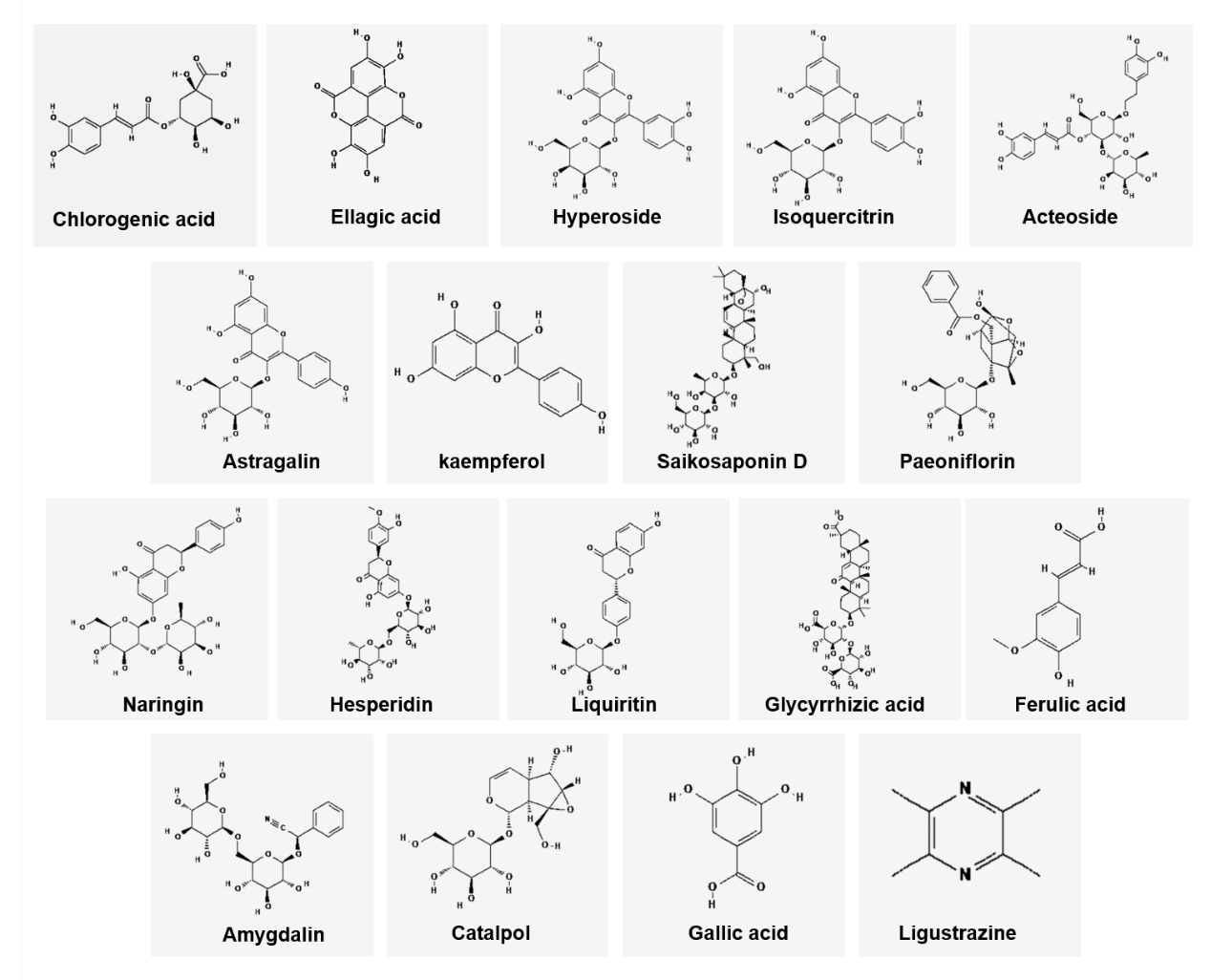
Table S3** List of ZYF-RIF intersection proteins

| **Gene**  **Symbol** | **Description** | **Gene-Cards**  **Disease Score** | **Gene-Cards**  **Disease GIFTS** | **Batman**  **ZYF Score** |
| --- | --- | --- | --- | --- |
| *AKT1* | AKT Serine/Threonine Kinase 1 | 51 | 0.9 | 48.00 |
| *ANXA1* | Annexin A1 | 48 | 0.63 | 48.00 |
| *AR* | Androgen Receptor | 51 | 0.81 | 122.78 |
| *ASPH* | Aspartate Beta-Hydroxylase | 42 | 0.66 | 122.78 |
| *BCL2* | BCL2 Apoptosis Regulator | 49 | 0.54 | 48.00 |
| *CALCA* | Calcitonin Related Polypeptide Alpha | 43 | 2.55 | 55.44 |
| *CD40LG* | CD40 Ligand | 46 | 0.66 | 122.78 |
| *CFTR* | CF Transmembrane Conductance Regulator | 50 | 0.71 | 48.00 |
| *CNR1* | Cannabinoid Receptor 1 | 46 | 0.68 | 48.00 |
| *CNR2* | Cannabinoid Receptor 2 | 45 | 0.55 | 48.00 |
| *CSF2* | Colony Stimulating Factor 2 | 42 | 1.39 | 55.44 |
| *CX3CR1* | C-X3-C Motif Chemokine Receptor 1 | 44 | 1.79 | 55.44 |
| *CXCR4* | C-X-C Motif Chemokine Receptor 4 | 50 | 1.94 | 48.00 |
| *DLL4* | Delta Like Canonical Notch Ligand 4 | 45 | 0.54 | 80.88 |
| *ESR1* | Estrogen Receptor 1 | 51 | 1.59 | 122.78 |
| *ESR2* | Estrogen Receptor 2 | 48 | 0.9 | 48.00 |
| *F2* | Coagulation Factor II, Thrombin | 48 | 2.19 | 80.88 |
| *GABRP* | Gamma-Aminobutyric Acid Type A Receptor Subunit Pi | 39 | 0.55 | 80.88 |
| *HLA-DQA1* | Major Histocompatibility Complex, Class II, DQ Alpha 1 | 42 | 0.54 | 55.44 |
| *IFNG* | Interferon Gamma | 47 | 1.63 | 48.00 |
| *IL10* | Interleukin 10 | 46 | 1.43 | 55.44 |
| *IL1B* | Interleukin 1 Beta | 46 | 5.43 | 55.44 |
| *IL2* | Interleukin 2 | 44 | 0.54 | 48.00 |
| *IL4* | Interleukin 4 | 44 | 0.54 | 686.00 |
| *IL5* | Interleukin 5 | 44 | 0.96 | 55.44 |
| *INS* | Insulin | 46 | 0.55 | 55.44 |
| *JUN* | Jun Proto-Oncogene, AP-1 Transcription Factor Subunit | 47 | 0.54 | 48.00 |
| *MAOA* | Monoamine Oxidase A | 48 | 0.88 | 48.00 |
| *MAPK1* | Mitogen-Activated Protein Kinase 1 | 50 | 0.84 | 122.78 |
| *MAPK3* | Mitogen-Activated Protein Kinase 3 | 48 | 0.55 | 80.88 |
| *MAPK8* | Mitogen-Activated Protein Kinase 8 | 48 | 0.54 | 48.00 |
| *MMP9* | Matrix Metallopeptidase 9 | 53 | 3.68 | 48.00 |
| *NFKB1* | Nuclear Factor Kappa B Subunit 1 | 50 | 1.79 | 80.88 |
| *PGR* | Progesterone Receptor | 48 | 4.08 | 122.78 |
| *PLAT* | Plasminogen Activator, Tissue Type | 48 | 0.83 | 48.00 |
| *PPARD* | Peroxisome Proliferator Activated Receptor Delta | 46 | 2.51 | 122.78 |
| *PPARG* | Peroxisome Proliferator Activated Receptor Gamma | 51 | 0.93 | 122.78 |
| *PRKCA* | Protein Kinase C Alpha | 49 | 0.73 | 48.00 |
| *PTGER2* | Prostaglandin E Receptor 2 | 48 | 1.55 | 48.00 |
| *PTGIR* | Prostaglandin I2 Receptor | 46 | 0.54 | 48.00 |
| *PTGIS* | Prostaglandin I2 Synthase | 46 | 0.54 | 80.88 |
| *PTGS2* | Prostaglandin-Endoperoxide Synthase 2 | 48 | 4.11 | 686.00 |
| *RAF1* | Raf-1 Proto-Oncogene, Serine/Threonine Kinase | 52 | 1.39 | 55.44 |
| *RELA* | RELA Proto-Oncogene, NF-KB Subunit | 49 | 0.63 | 122.78 |
| *RHOA* | Ras Homolog Family Member A | 46 | 0.73 | 55.44 |
| *SCNN1A* | Sodium Channel Epithelial 1 Subunit Alpha | 46 | 2.43 | 80.88 |
| *SLC18A2* | Solute Carrier Family 18 Member A2 | 46 | 0.78 | 48.00 |
| *SLC6A2* | Solute Carrier Family 6 Member 2 | 47 | 0.55 | 80.88 |
| *SLC6A4* | Solute Carrier Family 6 Member 4 | 47 | 1.57 | 48.00 |
| *THBD* | Thrombomodulin | 44 | 0.55 | 48.00 |
| *TNF* | Tumor Necrosis Factor | 50 | 3.41 | 80.88 |

**Table S4** Gene Ontology (GO) analysis of the biological process of ZYF-RIF

| **Term** | **Description** | **Log (FDR)** |
| --- | --- | --- |
| GO:0032496 | response to lipopolysaccharide | -20.07 |
| GO:0050727 | regulation of inflammatory response | -17.23 |
| GO:0006954 | inflammatory response | -17.00 |
| GO:0001934 | positive regulation of protein phosphorylation | -17.00 |
| GO:0044706 | multi-multicellular organism process | -13.79 |
| GO:0051090 | regulation of DNA-binding transcription factor activity | -13.61 |
| GO:0030335 | positive regulation of cell migration | -13.60 |
| GO:0050865 | regulation of cell activation | -13.60 |
| GO:2001234 | negative regulation of apoptotic signaling pathway | -13.27 |
| GO:0048660 | regulation of smooth muscle cell proliferation | -11.59 |
| GO:0009612 | response to mechanical stimulus | -10.85 |
| GO:0010632 | regulation of epithelial cell migration | -10.64 |
| GO:0048732 | gland development | -10.45 |
| GO:0071417 | cellular response to organonitrogen compound | -9.92 |
| GO:0055082 | cellular chemical homeostasis | -9.62 |
| GO:0036293 | response to decreased oxygen levels | -9.42 |
| GO:0048608 | reproductive structure development | -9.06 |
| GO:0000302 | response to reactive oxygen species | -8.66 |
| GO:0019233 | sensory perception of pain | -8.19 |
| GO:0043269 | regulation of ion transport | -7.92 |

**Table S5** ZYF-RIF intersection target protein gene action T cell-associated list

| **Term** | **Description** | **Log (FDR)** | **COUNT** | **Gene**  **Ratio** |
| --- | --- | --- | --- | --- |
| GO:0050870 | **positive regulation of T cell activation** | -7.75203 | 9 | 0.04 |
| GO:0050863 | regulation of T cell activation | -7.4962 | 10 | 0.03 |
| GO:0046637 | regulation of alpha-beta T cell differentiation | -5.24666 | 5 | 0.07 |
| GO:0046631 | alpha-beta T cell activation | -5.13788 | 5 | 0.07 |
| GO:0030217 | T cell differentiation | -4.99193 | 6 | 0.04 |
| GO:0042110 | T cell activation | -4.92858 | 7 | 0.03 |
| GO:0042129 | regulation of T cell proliferation | -4.64111 | 6 | 0.03 |
| GO:0045582 | positive regulation of T cell differentiation | -4.59636 | 5 | 0.05 |
| GO:0042102 | positive regulation of T cell proliferation | -4.43089 | 5 | 0.05 |
| GO:0046634 | regulation of alpha-beta T cell activation | -4.3942 | 5 | 0.05 |
| GO:0043370 | regulation of CD4-positive, alpha-beta T cell differentiation | -4.20393 | 4 | 0.08 |
| GO:0046632 | alpha-beta T cell differentiation | -3.97245 | 4 | 0.07 |
| GO:2000514 | regulation of CD4-positive, alpha-beta T cell activation | -3.77396 | 4 | 0.06 |
| GO:0045580 | regulation of T cell differentiation | -3.7146 | 5 | 0.03 |
| GO:0043371 | negative regulation of CD4-positive, alpha-beta T cell differentiation | -3.70505 | 3 | 0.14 |
| GO:0046639 | negative regulation of alpha-beta T cell differentiation | -3.50277 | 3 | 0.12 |
| GO:2000515 | negative regulation of CD4-positive, alpha-beta T cell activation | -3.2411 | 3 | 0.10 |
| GO:0046636 | negative regulation of alpha-beta T cell activation | -2.89572 | 3 | 0.07 |
| GO:0050868 | negative regulation of T cell activation | -2.8219 | 4 | 0.03 |
| GO:0045581 | negative regulation of T cell differentiation | -2.791 | 3 | 0.07 |
| GO:0045600 | positive regulation of fat cell differentiation | -5.24666 | 5 | 0.07 |
| GO:0045598 | regulation of fat cell differentiation | -5.10507 | 6 | 0.04 |

**Table S6** Enrichment analysis results of ZYF-RIF intersection target protein KEGG pathway

| Term | Description | Log (FDR) | COUNT | Gene Ratio |
| --- | --- | --- | --- | --- |
| **hsa04660** | **T cell receptor signaling pathway** | **-27.11** | **17** | **0.16** |
| hsa05330 | Allograft rejection | -13.67 | 8 | 0.21 |
| hsa05140 | Leishmaniasis | -18.51 | 12 | 0.16 |
| **hsa04658** | **Th1 and Th2 cell differentiation** | **-19.36** | **12** | **0.13** |
| hsa05030 | Cocaine addiction | -7.14 | 5 | 0.10 |
| hsa05418 | Fluid shear stress and atherosclerosis | -17.48 | 13 | 0.09 |
| hsa05120 | Epithelial cell signaling in Helicobacter pylori infection | -4.83 | 4 | 0.06 |
| hsa04211 | Longevity regulating pathway | -5.91 | 5 | 0.06 |
| hsa05200 | Pathways in cancer | -29.14 | 26 | 0.05 |
| hsa04721 | Synaptic vesicle cycle | -3.17 | 3 | 0.04 |
| hsa04728 | Dopaminergic synapse | -5.11 | 5 | 0.04 |
| hsa04610 | Complement and coagulation cascades | -3.07 | 3 | 0.04 |
| hsa04670 | Leukocyte trans endothelial migration | -4.02 | 4 | 0.04 |
| hsa05202 | Transcriptional mis regulation in cancer | -5.62 | 6 | 0.03 |
| hsa04080 | Neuroactive ligand-receptor interaction | -5.25 | 7 | 0.02 |

# STUDY OF OBJECTIVES

## Objectives

With the widespread use of TCM in vitro fertilization (IVF), studies with larger sample sizes need to evaluate the therapeutic efficacy of improving the infertility of RIF females. Furthermore, mechanisms of TCM multi-channel interventional in the changes of Th1/Th2, Treg in peripheral blood of RIF women are needed to verify.

## Endpoints

### The pregnancy outcomes after IVF-ET

The primary outcome was clinical pregnancy per woman, which was defined as the proportion of clinical pregnancy and the enrolled women. And clinical pregnancy is defined as the presence of at least one intrauterine gestational sac or fetal heartbeat confirmed by ultrasound, 4 ~ 6 weeks after embryo transfer or natural conception. Preceptificed second outcomes were the natural conception rate, conception rate, and pregnancy loss (defined as biochemical miscarriage, clinical pregnancy loss, ongoing pregnancy, and ectopic pregnancy).

Biochemical miscarriage (BM) is defined as only a chemical measure that indicates that pregnancy occurred, and there can be no evidence of a gestational sac detected on transvaginal ultrasound 2 weeks later. Therefore, in the definition of our trial, the parameters used to measure the hCG level differ substantially between > 5 to > 25 mIU/ml[[5](#_ENREF_11),[4](#_ENREF_12)]. And biochemical miscarriage rate is defined as the proportion of BM and all conception women (including the natural conception population). Ongoing pregnancy is controversial over studies, it is defined as the presence of one or more intrauterine fetuses with a positive heartbeat at 12 weeks gestational sonography in this trial[[1](#_ENREF_13)]. Clinical pregnancy loss is the opposite of ongoing pregnancy which is defined as the fetuses without a positive heartbeat until 12 weeks.

### The expression levels of Th1, Th2, and Treg in peripheral blood

Flow cytometry was used to detect the level of Treg cells (BD cytofix / cytoperm) in peripheral blood mononuclear cells ™ Fixation/permeabilization solution kit, (554714, BD company, CA, USA), CD4-FITC（eBioscience，11-0049-41, CD25-PE（eBioscience，12-0259-42）. The specific steps were strictly following the instructions of the kit.

### Maternal and child health during the perinatal period

The pregnancy and prenatal outcomes will be obtained by follow-up with the patient as per Society of Assisted Reproductive Technologies (SARTs) reporting guidelines. Maternal health indicators included Down’s syndrome screening (through nuchal translucency (NT), Oscar Test, noninvasive DNA examination, or amniocentesis), the incidence of pregnancy complications, which include placenta previa, oligohydramnios, premature rupture of membranes (PROM), gestational diabetes, gestational hypertension, intrahepatic cholestasis of pregnancy (ICP), and pregnancy anemia. Furthermore, delivery, postpartum condition, and related complications will be observed to evaluate maternal health. The neonatal outcomes (including basic characteristics of newborn, neonatal complications, and neonatal development)will be observed as follows up in 3 months and 1 year.

# STUDY DESIGN


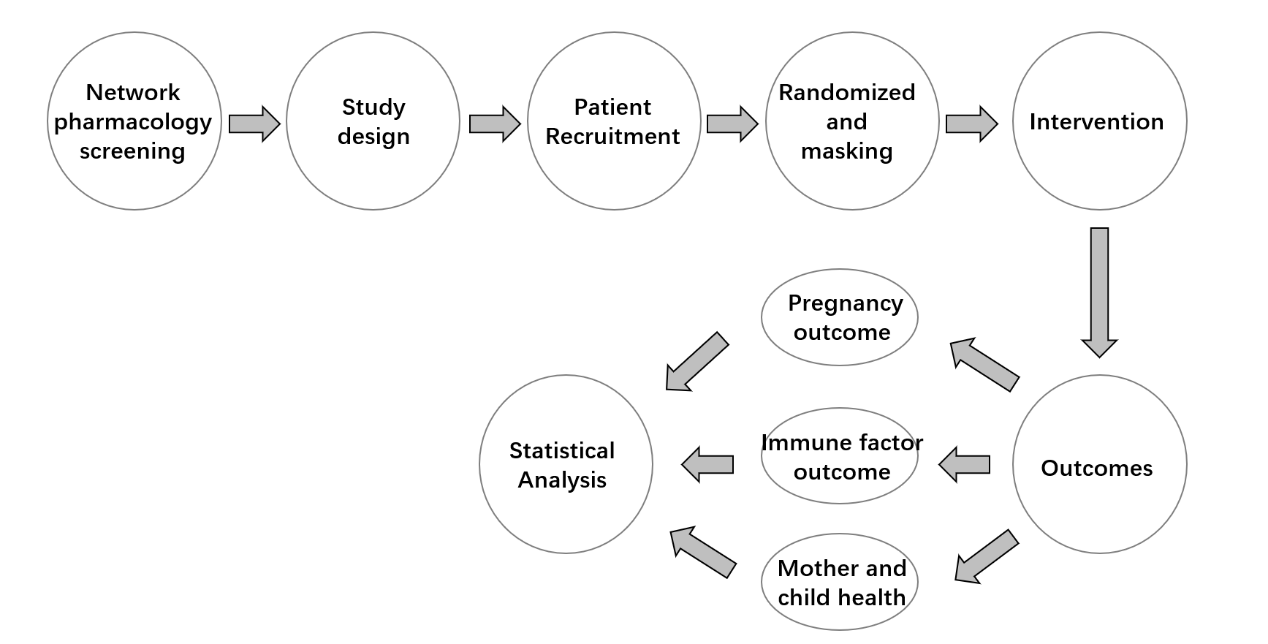
This trial is designed as a non-blinded, single-center, prospective randomized controlled trial during IVF treatment. The flow chart of the whole process of study shown in the Fig S2

**FIHURE S2:** The flow chart of the whole process of the study.

# STUDY POPULATION

## Number of participants

Combining pilot data, evidence from the systematic reviews, and clinical advice, we estimated that a 25 % or greater increase in the proportion of clinical pregnancy will be clinically important. To obtain 80% power at a 5% significance level for a 2-sided test, we assume a proportion of 24.5% clinical pregnancies in the control group and 49.5% clinical pregnancies in the treatment group. The minimal sample size calculated is 55 for each group. Considering about a 10% dropout rate, 121 participants in total are needed.

## Inclusion criteria

Though there is no universally agreed upon definition, RIF is often defined as the failure to achieve a clinical pregnancy after the repeated transfer (≥3) of four or more good-quality embryos in a woman under the age of 40 years[[1](#_ENREF_2)]. In this trial, participants diagnosed with RIF and aged between 20 ~ 39 years who were undergoing ART, will be included.

## Exclusion criteria

Women who were planning cycles of preimplantation genetic testing (PGT) were excluded from this trial, as well as those with a diagnosis of congenital abnormality (such as a submucous myoma, intrauterine adhesion, or uterine malformation), autoimmunity, endocrine disorders, and the husband was diagnosed with serious asthenozoospermia or oligospermia. We also excluded patients with liver disease or dysfunction, severe anemia, history of deep venous thrombosis, and an allergic constitution for IVF. Other severe diseases such as heart or kidney failure, previous cancer diagnosis, and pregnancy were excluded also.

# PARTICIPANT SELECTION AND ENROLMENT

## Identifying participants

The recruitment will include self-referral or referral from clinicians by advertisements or pamphlets on the internet. The recruitment strategy will include medical specialists and clinicians at the IVF units or TCM department.

## Screening for eligibility

Eligible women will be screened by the research nurses or trial coordinator not involved in the randomization process. Women meeting the criteria will be invited to consent to the trial on the day diagnosed with RIF. Women refusing randomization will be invited to join the standard care group.

## Consenting participants

Following consent, a case report form (CRF) will record information on reproductive, clinical, and demographic characteristics and will sign an informed consent form.

## Randomization

### Randomization

Randomization will be done into two study groups, the TCM multi-channel (ZYF and auricular acupuncture) group, and the control group. Women will be allocated to a study group by an internet randomization service in the sequence based on a computer-generated list.

### Treatment Allocation

Nurses or trial coordinators will do the randomization with the sealed envelope technique.

### Withdrawal procedures

If a participant desire to withdraw from the trial they can withdraw immediately. Women will be requested to complete the measurement at the nearest time point. If a subject withdraws during the intervention they will not be replaced. If they withdraw immediately the following randomization will be replaced.

# INTERVETION

## The CAM treatment group

The CAM treatment group will be administrated by two kinds of therapy: ZYF and auricular acupuncture.

### ***Zhuyun Formula***

ZYF contains Tu Sizi (*Cuscuta chinensis Lam.*) 15g, Fu Penzi (*Rubus idaeus L.)*, Gou Qizi (*Lyciumchinense Mill.)* 10g, Chai Hu (*Bupleurum chinense*) 10g, Bai Shao (*Cynanchum otophyllum*) 15g, Zhi Ke (*Poncirus trifoliata (L.) Raf*) 10g, Dang Gui (*Angelica sinensis (Oliv.) Diels*) 10g, Chuan Xiong (*Ligusticum chuanxiong Hort.*) 10g, Shu Dihuang (*Rehmannia glutinosa (Gaert.) Libosch. ex Fisch. et Mey.*) 10g, Zhi Gancao (*Glycyrrhiza uralensis Fisch.*) 5g. Each of the herbs was produced and packed in a single batch by China Resources Sanjiu Medical and Pharmaceutical Co., Ltd., Shenzhen, China. On taking, mix the herbs with hot water orally third times daily for 3 months before IVF/ICSI, 200 ml per time. The details of the components were listed below. (see Table S1).

### Auricular acupuncture


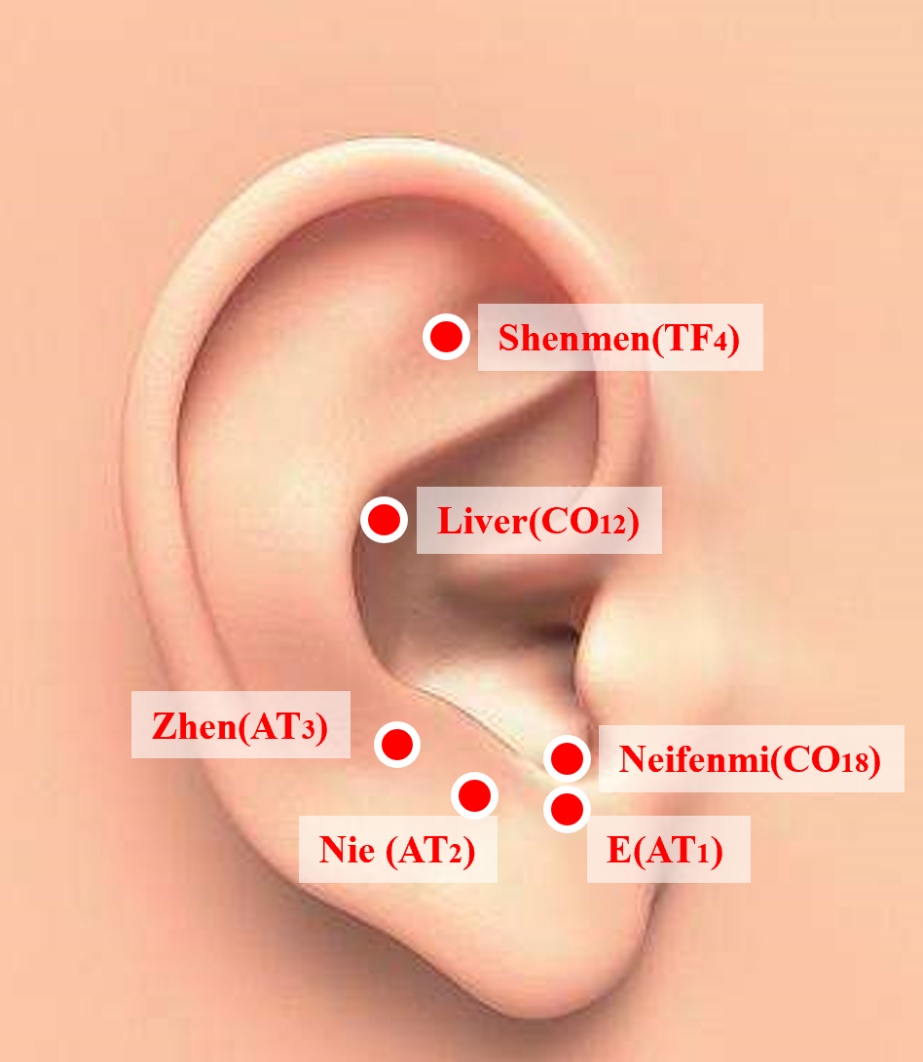


**FIGURE S3**: Location of Auricular point.

Small stainless needles for auricular treatment at the following points, Liver (CO 12), Shenmen (TF4), Neifenmi (CO_18_), E (AT1), Nie (AT2), Zhen (AT3) were used in the trial before IVF/ICSI for three months, once per week. All treatments were performed by the same well-trained examiner in the same way (see **Fig. S3)**.

## The control group

In the control group: all participants were naturally waiting for 3 months before IVF/ICIS.

## IVF ovarian stimulation protocol

All subjects were undergoing ART after three months, standard long agonist protocol for ovarian stimulation will be performed[[4](#_ENREF_16)]. Egg retrieval, fertilization, and embryo transfer will be determined by their treating clinician.

# SAMPLE COLLECTION

Serum levels of Th1, Th2, and Treg were measured before and after the intervention at the middle luteal phase (6 ~7 days after LH peak, progesterone ≥ 5 ng/ml). 4 ml of complete blood was collected by vacuum blood collection vessel anticoagulant with heparin sodium. The monocytes in peripheral blood were isolated and cultured. The samples were stored in a 4 ℃ refrigerator and analyzed within 24 hours.

# DEFINITION OF OUTCOMES

**Table S7** The definition of the pregnancy outcomes

| **Items** | **Definition** |
| --- | --- |
| Clinical pregnancy | detection of a gestational sac in the uterine cavity |
| Natural pregnancy | natural pregnancy without IVF-ET |
| Conception | serum human chorionic gonadotropin ≥10 mIU/mL |
| Ongoing pregnancy | detection of a viable fetus with fetal heartbeat at 11–12 weeks’ gestation. |
| Biochemical miscarriage | serum human chorionic gonadotropin ≥ 25 mIU/mL without gestational sac |
| Clinical pregnancy loss | including biochemical miscarriage and clinical pregnancy lost |

# STUDY ASSESSMENTS

## Safety assessments

Any adverse events or reactions associated with the intervention will be recorded on the CRF and reported to the principal investigator Qian Zeng. The investigator will exercise her scientific judgment on whether an abnormal finding or other abnormal assessment is clinically significant. All adverse events reported between consent and final follow-up will be recorded on the adverse event page. A safety monitoring committee will be convened to examine events.

The perinatal and neonatal outcomes will be collected by follows up. The perinatal outcomes include: the way of Down’s syndrome-related screening (nuchal translucency, Oscar Test, noninvasive DNA examination, and amniocentesis). Pregnancy-related complications and idiopathic diseases (placenta previa, oligohydramnios, premature rupture of membranes (PROM), gestational diabetes, gestational hypertension, intrahepatic cholestasis of pregnancy (ICP), pregnancy anemia, thrombocytopenia during pregnancy, and pregnancy with thyroid disease). Delivery, postpartum condition, and related complications (gestational week of delivery, vaginal delivery, cesarean section, adherent placenta, abnormal lochia, oligogalactia,). The neonatal outcomes include basic characteristics of newborn (birth weight, birth height, number of birth deformity, number of apgar score ＜ 7), neonatal complications (jaundice of the newborn, pneumonia of the newborn, hypoglycemia of the newborn), neonatal development (weight after 3 months, weight after 1 year, height after 3 months, height after 1 year, history of pneumonia, history of diarrhea, history of other diseases).

## Study assessments

Characteristics will be assessed at the baseline. The primary outcome and the second outcomes will be investigated at 2 and 12 weeks. Clinical outcome data will be collected following the blood hCG test, clinical ultrasound, and ongoing pregnancy. Maternal health indicators include basic information of newborn birth, adverse birth rate, disease status of newborn, length and weight change of newborn after 3 months and 1 year.

# DATA COLLECTION

The research nurse/coordinator employed at the IVF Unit will be responsible for data collection of the reproductive outcomes. This data is collected routinely by all IVF units. To ensure data collection on live births is timely, we will seek the women’s permission to contact her following her expected date of delivery. Contact details will be collected from the woman and nominated contacts to advise on change of address, three monthly phone calls will be made to maximize retention and loss to follow up, this will be done by the trial coordinator.

# EXPERIMENTAL METHODS

## Laboratory reagent

BD Cytofix/Cytoperm™ Fixation/Permeabilization Solution Kit， intracellular antigen staining membrane breaker; BD FACS Lysing Solution(hemolysin); BD heparin sodium green cap blood vessel 6ml; PBS, pH 7.4，（Gibco）; Fetal Bovine Serum， Gibco; RPMI 1640 cell culture medium (hyclo); Human total Foxp3 protein (Foxp3) ELISA Kit (Shanghai Xintang Biotechnology Co., Ltd); ReverTra Ace qPCR Kit (TOYOBO, FSQ-101); Power SYBR Green PCR Master Mix (ABI, 4368708); Nuclease-Free Water (not DEPC-Treated) (Ambion, Cat# AM9937); 384-well Optical Reaction Plate with barcode (ABI, Cat. #4309849); MicroAmp Optical Adhesive Film (ABI, Cat. #4311971).

## Experimental instruments and equipment

Microplate Reader, DENLEY DRAGON Wellscan MK 3, Finland Leibo; Laundry board, Wellwash 4 MK2, Finland Leibo; Vortex mixer, XW-80A, Shanghai Qingpu Huxi Instrument Factory; Incubator, DHP-9052, Thermo Revco; Tabletop centrifuge, TD-60B； Autoclave pans, YX-280, 18L; Continuous separators, 0.1～50ml, TOMOS LIFE SCIENCE Group; High throughput tissue grinders, Auto-Pure20A, Ningbo Xinzhi; Flow cytometry, FACSCanto II， BD; CO2 incubator, BBD 6220, Thermo Scientific; Type B2 biological safety cabinet, 1300 Series II, Thermo Scientific; Multipurpose vortex mixer, SI Vortex-Genie2, American Scientific; Medical nucleic acid molecular rapid hybridisers, Kemp hhm-2; DNA amplimers, Zhuhai HEMA company; Electric blast drying oven, 101-1, Jiangsu Dongtai; Ultra clean bench, TDGL2J, Suzhou voltage regulating electric appliance factory; Centrifuge tube, Falcon; Sample loading pipette tip, Shanghai Qiujing biochemical reagent Instrument Co., Ltd.

## Patient peripheral blood sample collection steps

After the patients in both groups met the inclusion criteria, they signed the informed consent to aseptically collect 10 ml of peripheral blood from arm vein, use heparin anticoagulant tube (green tube), shake gently to make the anticoagulant dissolve sufficiently; After sufficient dissolution, the same volume of room temperature physiological saline was added into a sterile tube to make the blood dilute evenly, reduce erythrocyte aggregation and improve its separation effect; Lymphocyte layering fluid (3 to 5 ml per 10 ml of diluted blood) was placed into a 15 ml centrifuge tube, which was then tilted 45 degrees, and the diluted blood was slowly added to the above layering fluid along the tube wall from the layering fluid interface, taking care to maintain clarity between the interfaces and not mixing the blood into the layering fluid, otherwise it was counted as a failure; The tubes were placed in a horizontal centrifuge and centrifuged at 3000 rpm for a total of 20 min at 18 to 20 ° C. One ml pipette tip was gently inserted into the gray layer and the gray-white mononuclear cells were gently aspirated along the wall into another centrifuge tube; Wash the PBMC suspension 2 times with 5 volumes of NS and centrifuge at 1500 rpm for a total of 10 min at room temperature.

## Procedure for CD4^+^CD25^+^Foxp3 detection

Take the flow loading tube and add 20 UL each of cd4-fitc (ebioscience, 11-0049-41), cd25-pe (ebioscience, 12-0259-42) monoclonal antibodies or corresponding isotype controls sequentially to the bottom of the tube; 100 UL of peripheral blood after mixing was added; Incubate for 30min at room temperature in the dark; Add 1 ml Foxp3 fix/perm (ebioscience) working solution to each tube, mix well, and incubate at 4 degrees for 30-60 min; Add 2 ml of 1 * perm buff per tube, centrifuge at 350g for 5 min, pour off the supernatant and resuspend the cells in 100 UL of 1 * perm buff; Foxp3 APC (ebioscience, 17-5777-82) and corresponding isotype control antibodies were added and incubated for 30-60min in the dark at room temperature; Add 2 ml of 1 * perm buff, centrifuge at 350g for 5 min, and pour off the supernatant; Repeat step 7 once; Add 500 UL PBS, mix and then apply to the machine for detection. If it could not be detected immediately, PBS was added to a final concentration of 1% paraformaldehyde for storage and tested within 24 h.

## Th1, Th2 expression (CD4 ^+^IFN-r^+^IL-4^+^) detection steps

A 100 UL specimen of whole blood was taken, diluted in equal volume with RPMI 1640 1:1 (hyclo), and then divided into 2 parts to add into a sterile tube, labeled as an unstimulated tube (tube a) and a stimulated tube (tube B). If with PBMC specimens, then isolate mononuclear cells with lymphocyte separation solution, then take 200ul of PBMC (2 ~ 10 x 106 cells / ml) for experiments; A tube was added with a final concentration of 10 UG / ml BFA solution (brefeldin A, a protein transport inhibitor) and B tube was added with a final concentration of 50 ng / ml PMA (phorbol ester) + 1 ug / ml ionomycin (ionomycin) + 10 UG / ml BFA solution, mixed, and incubated at 37 ° C in a 5% CO2 incubator for 4-6 hours; PBS wash cells one time; Mix the cells and add 20 UL of CD4 - percp CY to each tube ™ 5.5 (BD, 552838), mix, and incubate for 30 min in the dark at room temperature; Add 100 UL of reagent A (i.e., fixative) in fix & perm (BD) per tube and incubate for 5 minutes at room temperature, protected from light, then add 2 ml of 1 × BD FACS lysing solution and incubate for 10 minutes protected from light; Centrifuge at 500g for 5 min and discard the supernatant; Separate each tube by 50 μ L membrane disruptor, IFN- γ- FITC (cat. No. BD, 561057) and il-4-pe (cat. No. BD, 555082) antibodies or homologous control were incubated for 30 min; 4 ml PBS was added into each tube and centrifuged at 350g for 5 min, the supernatant was discarded, 500 UL PBS was added and the mixture was followed by upper machine detection. If it could not be detected immediately, PBS was added to a final concentration of 1% paraformaldehyde for storage and tested within 24 h.

## Foxp3 protein detection steps

Samples were diluted 1:10 fold with distilled water. The serum was stored frozen (- 20 ° C). After collecting the sample, the next step was taken to avoid repeated freeze-thawing; Standard solutions were prepared; A 100.0 μ L the sample to be tested (activated) is added to each well and the reaction plate is thoroughly mixed and placed at 37 ° C for a total of approximately 40 min; Washing the plate: the reaction plate was thoroughly washed 4-6 times with washing solution and dried on filter paper; Add 50 UL distilled water and primary antibody working solution per well (except blank). The reaction plate was mixed well and placed at 37 ° C for 20 min, and the wash plate was treated the same as the previous step; Add 100 μ L of enzyme-labeled antibody working solution in each well. The reaction plate was placed at 37 ° C for 10 min. Washing the plates was treated the same as in the previous step; To each well was added 100 μ μ l of substrate working solution and allowed to react for 15 min at 37 ° C in dark light. Add 100 UL of stop solution to each specimen well to mix, and measure the absorbance at 450 nm with a microplate reader over 930 min; After subtracting blank values, all OD values should be calculated again. (if blank od < 0.1, calculated directly); Standard curves were plotted using masterplex readerfit with abscissa for standards: 2000, 1000, 500, 250, 125, 62.5, 31.2, 0 in pg/ml and ordinate: OD; The corresponding Foxp3 content values were derived from the - OD values of the specimens, and finally multiplied by the dilution factor, remembering the FOXP3 protein expression content in pg /ml.

# STATISTICS AND DATA ANALYSIS

To assess the effect of missing data, an intention-to-treat analysis was planned and we performed post-hoc sensitivity analyses, fitting best- and worst-case scenarios. For the best-case scenario, we assumed all unknown events in the treatment group were positive (clinical pregnancy) and those in the control group were negative. For the worst-case scenario, we assumed none of the women with missing data in the treatment group became pregnant, and all of the women with missing data in the control group did become pregnant.

Spss25.0 statistical software was used for statistics, and the measurement data was expressed as ‾X ± s, The data were normal distribution, the independent sample t-test was used for intergroup comparison, and paired sample t-test was used for intragroup comparison; if the data were skew distribution, Mann Whitney U test was used between groups, Wilcoxon signed rank-sum test, case/control association analysis and chi-square test were used before and after the group, and the difference was statistically significant (P < 0.05).

# MONITORING AND QUALITY ASSURANCE

A monthly management teleconference will be taken by the researchers to examine trial recruitment accrual, data quality, compliance with the protocol, and organization and implementation of the trial protocol.

# ETHICAL CONDUCT OF THE STUDY

The study was approved by the ethics committees (2016KL-013). The authors registered the trial with Clinical-Trials.gov (NCT03078205)

# REFERENCES

[1] Oral S., Karacan M., Akpak Y.K., et al. Live birth rate with double ovarian stimulation is superior to follicular phase ovarian stimulation per started cycle in poor ovarian responders. The journal of obstetrics and gynaecology research. 2021,47(8):2705-12 doi: 10.1111/jog.14871

[2] Bashiri A., Halper K.I., Orvieto R. Recurrent Implantation Failure-update overview on etiology, diagnosis, treatment and future directions. Reproductive biology and endocrinology : RB&E. 2018,16(1):121 doi: 10.1186/s12958-018-0414-2

[3] Comins Boo A., Segovia A.G., Del Prado N.N., et al. Evidence-based Update: Immunological Evaluation of Recurrent Implantation Failure. Reproductive Immunology: Open Access. 2016,01(04) doi:

[4] Smith C.A., De Lacey S., Chapman M., et al. Effect of Acupuncture vs Sham Acupuncture on Live Births Among Women Undergoing In Vitro Fertilization: A Randomized Clinical Trial. Jama. 2018,319(19):1990-8 doi: 10.1001/jama.2018.5336

[5] Yang G.Y., Luo H., Liao X., et al. Chinese herbal medicine for the treatment of recurrent miscarriage: a systematic review of randomized clinical trials. BMC complementary and alternative medicine. 2013,13:320 doi: 10.1186/1472-6882-13-320

[6] Sun J., Song J., Dong Y., et al. Erzhi Tiangui Granules Improve In Vitro Fertilization Outcomes in Infertile Women with Advanced Age. Evidence-based complementary and alternative medicine : eCAM. 2021,2021:9951491 doi: 10.1155/2021/9951491

[7] Zhimin L.Y.-j.A.M.-y.P.Y.D.B.-h.J.Y.-x.H.C.-j.C. Effect of Salt-water Processing on the Contents of Main Chemical Constituents in Wuzi Yanzong Pills. Journal of Chinese Medicinal Materials. 2021,44(08):1851-5 doi:

[8] Yuefeng W.H.L.D.Z.Y.Z.S.N.J.S.X.L. Research Progress of Sini Powder and Prediction Analysis on Q-markers. Chinese Archives of Traditional Chinese Medicine. 2021,39(12):13-20 doi:

[9] Jinming; N.X.C.Y.-f.W.L.F.C.-m.H.Y.Z. Review of Chemical Constituents,Pharmacological Effects and Clinical Applications of Taohong Siwutang and Predictive Analysis of Its Quality Marker. Chinese Journal of Experimental Traditional Medical Formulae. 2020,26(04):226-34 doi:

[10] Cao L., Chen H., Huang Y., et al. The Pharmacological Activity of the Wenjing Decoction in Recurrent Spontaneous Abortion. Evidence-based complementary and alternative medicine : eCAM. 2021,2021:8861394 doi: 10.1155/2021/8861394

[11] Coulam C.B., Roussev R. Chemical pregnancies: immunologic and ultrasonographic studies. American journal of reproductive immunology (New York, NY : 1989). 2002,48(5):323-8 doi: 10.1034/j.1600-0897.2002.01137.x

[12] Zeadna A., Son W.Y., Moon J.H., et al. A comparison of biochemical pregnancy rates between women who underwent IVF and fertile controls who conceived spontaneously†. Human reproduction (Oxford, England). 2015,30(4):783-8 doi: 10.1093/humrep/dev024

[13] Brandes M., Hamilton C.J., De Bruin J.P., et al. The relative contribution of IVF to the total ongoing pregnancy rate in a subfertile cohort. Human reproduction (Oxford, England). 2010,25(1):118-26 doi: 10.1093/humrep/dep341

[14] Qianhong M.A., Shangwei L.I., Huang Z. Timing of Start of Controlled Ovarian Hyperstimulation after Pituitary Down-regulation by Depot GnRH Agonist. Journal of Practical Obstetrics and Gynecology. 2006 doi:

[15] Sun Y., Zhang Y., Ma X., et al. Determining Diagnostic Criteria of Unexplained Recurrent Implantation Failure: A Retrospective Study of Two vs Three or More Implantation Failure. Frontiers in Endocrinology. 2021,12 doi: 10.3389/fendo.2021.619437

[16] Medicine P.C.o.S.f.A.R.T.P.C.o.A.S.f.R. Elective single-embryo transfer. Fertility and sterility. 2012,97(4):835-42 doi: 10.1016/j.fertnstert.2011.11.050
